# Supplementary material for: Prostate-specific antigen (PSA) testing of men in UK general practice: a 10-year longitudinal cohort study
Source: BMJ Open. 2017 Oct 30;7(10):e017729. doi: 10.1136/bmjopen-2017-017729 (PMC5665300; doi:10.1136/bmjopen-2017-017729)
Supplement: Supplementary file 1 [file bmjopen-2017-017729supp001.pdf]

Supplementary material

Table S1. List of used medical codes for prostate cancer biopsies and diagnoses

**Biopsies**		**Diagnoses**	
Medcode	Readterm	Medcode	Readterm
1069	*Transrectal needle biopsy of prostate*	780	*Malignant neoplasm of prostate*
7908	*Open biopsy of prostate*	6328	*Carcinoma in situ of prostate*
7909	*Unspec diagnostic cystoscopic exam bladder & biopsy prostate*	10178	*Gleason grading of prostate*
12391	*Transurethral biopsy prostate*	18503	*Gleason prostate grade 2-4 (low)*
22297	*Trucut transperineal biopsy of prostate*	18612	*Gleason prostate grade 5-7 (medium)*
22473	*Transperineal needle biopsy of prostate*	26081	*Gleason prostate grade 8-10 (high)*
22719	*Endoscopic punch biopsy of prostate*	37306	*Personal history of malignant neoplasm of prostate*
		102314	*History of prostate cancer*

Table S2. The percentage of men diagnosed with prostate cancer in CPRD based on their first and second PSA test

**% diagnosed (n/N)**	**Second test (within 1 year) PSA level**[~]				
	No 2nd test	PSA<3	3≤PSA<6	6≤PSA<10	PSA≥10
**First test PSA level**[~]					
PSA<3	<1% (106/59883)	<1% (33/7315)	1% (4/413)	10% (7/70)	15% (7/46)
3≤PSA<6	3% (234/8133)	1% (6/748)	5% (162/3027)	14% (66/483)	15% (8/54)
6≤PSA<10	24% (452/1845)	1% (3/230)	4% (23/622)	17% (269/1556)	18% (45/249)
PSA≥10	61% (1232/2030)	6% (11/183)	3% (6/227)	9% (29/338)	43% (552/1281)

[~]Men with PSA tests without a level recorded are not included
