# Supplementary material for: Prostate-specific antigen (PSA) testing of men in UK general practice: a 10-year longitudinal cohort study
Source: BMJ Open. 2017 Oct 30;7(10):e017729. doi: 10.1136/bmjopen-2017-017729 (PMC5665300; doi:10.1136/bmjopen-2017-017729)
Supplement: Author's manuscript [file bmjopen-2017-017729.draft_revisions.pdf]

BMJ Open

BMJ Open is committed to open peer review. As part of this commitment we make the peer review history of every article we publish publicly available.

When an article is published we post the peer reviewers' comments and the authors' responses online. We also post the versions of the paper that were used during peer review. These are the versions that the peer review comments apply to.

The versions of the paper that follow are the versions that were submitted during the peer review process. They are not the versions of record or the final published versions. They should not be cited or distributed as the published version of this manuscript.

BMJ Open is an open access journal and the full, final, typeset and author-corrected version of record of the manuscript is available on our site with no access controls, subscription charges or pay-per-view fees (http://bmjopen.bmj.com).

If you have any questions on BMJ Open's open peer review process please email editorial.bmjopen@bmj.com

BMJ Open

Prostate Specific Antigen (PSA) testing of men in UK general practice: a 10-year longitudinal cohort study

Journal:	*BMJ Open*
Manuscript ID	bmjopen-2017-017729
Article Type:	Research
Date Submitted by the Author:	12-May-2017

Complete List of Authors:	Young, Grace; University of Bristol, School of Social and Community Medicine; Bristol Randomised Trials Collaboration, University of Bristol<br>Harrison, Sean; University of Bristol School of Social and Community Medicine<br>Turner, Emma; University of Bristol, School of Social and Community Medicine<br>Walsh, Eleanor; University of Bristol, School of Social and Community Medicine<br>Oliver, Steven; University of York, The Hull York Medical School<br>Ben-Shlomo, Yoav; University of Bristol,<br>Evans, Simon; Royal United Hospital, Urology Department<br>Lane, Athene; University of Bristol, School of Social and Community Medicine; Bristol Randomised Trials Collaboration, University of Bristol<br>Neal, David; University of Oxford Nuffield Department of Surgical Sciences<br>Hamdy, Freddie; University of Oxford Nuffield Department of Surgical Sciences<br>Donovan, Jenny; University of Bristol School of Social and Community Medicine<br>Martin, Richard; University of Bristol, Social Medicine<br>Metcalfe, Chris; University of Bristol, Social Medicine; Bristol Randomised Trials Collaboration, University of Bristol

**Primary Subject Heading**:	Public health
Secondary Subject Heading:	Diagnostics, Epidemiology, Urology
Keywords:	PSA testing, screening, primary health care, prostate cancer

SCHOLARONE™
Manuscripts

Prostate Specific Antigen (PSA) testing of men in UK general practice: a 10-year longitudinal cohort study

Running title: PSA testing in UK general practice: 2002-2012

Ms Grace J Young[1,2]* MSc, Mr Sean Harrison[1]* MA (Cantab), Dr Emma L Turner[1] PhD, Ms Eleanor I Walsh[1] MSc, Dr Steven E Oliver[3] PhD, Prof Yoav Ben-Shlomo[1] PhD, Mr Simon Evans[4] MRCS, Dr J Athene Lane[1,2] PhD, Prof David E Neal[5] FMedSci, Prof Freddie C Hamdy[5] FMedSci, Prof Jenny L Donovan[1] PhD, Prof Richard M Martin[1] PhD, Dr Chris Metcalfe[1,2] PhD

*denoting joint first authorship

[1]Address: School of Social and Community Medicine, University of Bristol, Canynge Hall, 39 Whatley Road, Bristol, BS8 2PS, UK

[2]Address: Bristol Randomised Trials Collaboration, University of Bristol, Canynge Hall, 39 Whatley Road, Bristol, BS8 2PS, UK

[3]Address: The Hull York Medical School, University of York, Heslington, York, YO10 5DD, UK

[4]Address: Urology Department, Royal United Hospital, Combe Park, Bath BA1 3NG, UK

[5]Address: Nuffield Department of Surgical Sciences, University of Oxford, Oxford, OX3 7DQ, UK

Correspondence: Dr Chris Metcalfe, email: chris.metcalfe@bristol.ac.uk, telephone: 0117 928 7326, address: School of Social and Community Medicine, University of Bristol, Canynge Hall, 39 Whatley Road, Bristol, BS8 2PS, UK

Funding: Supported by Cancer Research UK and the UK Department of Health for the CAP trial (C11043/A4286, C18281/A8145, C18281/A11326, and C18281/A15064). The ProtecT trial is funded by the UK National Institute for Health Research (NIHR), Health Technology Assessment Programme (projects 96/20/06, 96/20/99).

Word count: 4001

For peer review only
For peer review only - http://bmjopen.bmj.com/site/about/guidelines.xhtml

ABSTRACT

Objectives*:* Cross-sectional studies suggest that around 6% of men undergo PSA testing each year in UK general practice. This longitudinal study aims to determine the cumulative testing pattern of men over a 10-year period and whether this testing can be considered equivalent to screening for prostate cancer.

Setting, participants and outcome measures*:* Patient-level data on PSA tests, biopsies and prostate cancer (PCa) diagnoses were obtained from the UK Clinical Practice Research Datalink (CPRD) for the years 2002 to 2011. The cumulative risks of PSA testing and of being diagnosed with PCa were estimated for the 10-year study period. Associations of a man's age, region and index of multiple deprivation (IMD) with the cumulative risk of PSA testing and PCa diagnosis were investigated. Rates of biopsy and diagnosis, following a high test result, were compared to those from the programme of PSA testing in the ProtecT study.

Results*:* The 10-year risk of exposure to at least one PSA test in men aged 45 to 69 years in UK general practice was 39.2% (95% C.I. 39.0, 39.4%). The age-specific risks ranged from: 25.2% for 45-49 year olds to 53.0% for 65-69 year olds (P for trend<0.001). For those with a PSA level ≥3, a test in UK general practice was less likely to result in a biopsy (6%) and/or diagnosis of prostate cancer (15%) compared to ProtecT study participants (85% and 34% respectively).

Conclusion*:* A high proportion of 45-69 year old men undergo PSA tests in UK general practice: 39% over a ten year period. A high proportion of these tests appear to be for the investigation of lower urinary tract symptoms, and not screening for prostate cancer.

Trial registration: The ProtecT trial is registered at Current Controlled Trials (ISRCTN20141297) and Clinical Trials.Gov (NCT02044172).

For peer review only - http://bmjopen.bmj.com/site/about/guidelines.xhtml

STRENGTHS AND LIMITATIONS OF THIS STUDY

- This is the first study in the UK to look at patterns of PSA testing over a ten-year period in a cohort of men.
- Data on over 430,000 men could be analysed from the Clinical Practice Research Database and compared to data on 58,500 men from the programme of PSA testing and diagnostic biopsy in the ProtecT study

- The completeness of some routine data items is uncertain; with the recorded diagnoses outnumbering the recorded biopsies indicating that the latter are under-recorded
- It was not possible to distinguish tests undertaken in men with and without symptoms therefore the proportion of tests prompted by the presentation of LUTS was inferred.

For peer review only

For peer review only - http://bmjopen.bmj.com/site/about/guidelines.xhtml

INTRODUCTION

The UK currently runs three screening programmes for breast, bowel and cervical cancers. Prostate cancer is now the most commonly diagnosed cancer in men in the UK despite there being no formal screening programme.[1] Prostate Specific Antigen (PSA) level can be used as a screening test, with prostate biopsy in men with a raised PSA level allowing histopathological confirmation of the diagnosis of prostate cancer. Despite almost 30 years of PSA testing, the balance of benefits and harms of the test has not been established and, perhaps as a consequence, there are varying rates of testing around the UK and the world.[2] There is evidence that a PSA-based screening programme will reduce mortality due to prostate cancer[3] but with a risk of over diagnosis, such that a man diagnosed with cancer localised to the prostate would not have developed clinical symptoms of the disease in his lifetime if left untreated.[4 5] Radical treatment of such men exposes them to the risk of treatment-related adverse events without the potential to benefit.[6]

Current guidance for Primary Care Physicians in the UK, US and Australia recommends discussing and coming to a shared decision about PSA testing,[7] with men who either raise the issue or warrant consideration of testing, due to a family history of the disease for example. With such passive advice, variable testing rates across GPs are unsurprising. Three cross sectional studies have been conducted giving an indication of the PSA testing rates in the UK between 2001 and 2011. Melia et al., studying 469,159 men aged 45 to 84 years, reported an annual rate of 6% over 1999-2002 for England and Wales, with an annual rate of 2% in the absence of symptoms.[8] Williams et al., studying 126,716 men aged 45 to 89 years and without a prior diagnosis of prostate cancer, found 6.2% of these men received a PSA test during 2007.[9] This study concluded that testing was more prevalent in older men, more southern areas of the UK (especially Wales) and areas of lower deprivation. Moss et al. obtained data from the Clinical Practice Research Datalink on 650,264 men aged 45 to 84 and found a testing rate of 8.74 and 9.45 per 100 person-years in 2010 and 2011 respectively.[10] Again, rates increased with age and areas of lower deprivation. Of 49,306 men tested in 2010 and with at least 9 months of follow-up, 0.2% with a PSA level <3ng/ml were diagnosed with prostate cancer within 9 months, rising to 14.5% of men with PSA level >5ng/ml. A London-based study of 150,481 men aged 40

For peer review only - http://bmjopen.bmj.com/site/about/guidelines.xhtml

years or older found that 8.2% of men were PSA tested at their General Practice in the 12 months from August 2013 to July 2014.[11]

When PSA tests are undertaken for screening, men with a raised level will be referred for biopsy, with examination of prostate tissue necessary for the diagnosis of prostate cancer. Furthermore, as screen-detected prostate cancer is relatively slow to progress, screening is targeted at men in their fifties and sixties, the balance of risks of short-term treatment harms and longer-term survival benefit being less favourable for older men as death due to other causes is more likely and radical treatments less suitable. Tests which are unlikely to be followed-up by biopsy, and which are undergone by older men, are likely to be guiding the treatment of benign hyperplasia of the prostate.[12] Guidance for the assessment of lower urinary tract symptoms (LUTS), affecting approximately 30% of over 50s,[13] includes consideration of a PSA when LUTS are suggestive of bladder outlet obstruction secondary to benign prostatic enlargement; where PSA>1.4ng/ml can direct drug treatment decisions.[14]

While estimates of the number of men undergoing a PSA test in a twelve-month period give an indication of how widespread use of the test has become in UK general practice, a longitudinal perspective is needed to examine how the PSA test is being used to manage the risk of prostate cancer in individual men. Long term retrospective cohort studies of PSA testing rates have been conducted elsewhere in Europe;[15 16] however, the cumulative risks of PSA testing in the UK are yet to be quantified.

The primary objective of this study was to estimate the cumulative risk of PSA testing of UK men in primary care, without a diagnosis of prostate cancer, over the 10-year period 1st January 2002 to 31st December 2011. The association of testing rates with age, region and index of multiple deprivation (IMD) was investigated. The proportion of tests resulting in a biopsy and/or diagnosis of prostate cancer was compared to the programme of PSA testing, akin to screening, in the ProtecT study[17] to gauge whether PSA tests undertaken in UK General Practice can be considered as an effective attempt at screening.

For peer review only - http://bmjopen.bmj.com/site/about/guidelines.xhtml

SUBJECTS AND METHODS

Design

We undertook a retrospective cohort study of 450,000 men using data from the Clinical Practice Research Datalink (CPRD), a large primary care database.[18] The CPRD contains electronic medical records for approximately 4.4 million active patients in 674 practices, representing 6.9% of the UK population. Patients in the database were shown to be representative of the UK population in terms of age, sex, ethnicity and BMI. However, the data do not include prisoners, private patients, some residential homes and the homeless.[19] Practices participating in the CPRD have been found to have a greater number of patients compared to the national average.[20]

Data were requested for General Practice (GP) surgeries in all areas of UK, but excluding London as it is thought that PSA testing rates would be markedly different in the capital.[11] We included practices which contributed acceptable ‘research standard’ data for the observation period, 1st of January 2000 - 31st December 2011. Data requested from the CPRD included: age, Index of Multiple Deprivation (IMD) from 2004, region, GP practice size, mortality date and cause, occurrence of PSA tests and prostate biopsies. PSA test dates before 2002 were also collected to estimate how many of the men had received a test prior to registration. The Index of Multiple Deprivation (IMD) is an area based deprivation measure which ranges from 0 to 100 with higher scores indicating higher levels of deprivation. CPRD base these on the patients’ postcode (English residents only) and then create twentiles to ensure concealment of individuals’ place of residence.

Study population

Entry to the cohort commenced on the 1st January 2002. Person-years for the time before the first PSA test were calculated having censored men from the analysis at the earliest of: (1) the end of the study period (31st December 2011); (2) after receiving a prostate cancer diagnosis; or (3) death or transfer out of the practice. Men aged 45 to 69 at study entry were included (those born between 1933 and 1957).

Practices thought to be involved with research involving practice-wide PSA testing within the eligible age group were excluded. For example, the ProtecT study[17] was recruiting at UK general practices

For peer review only - http://bmjopen.bmj.com/site/about/guidelines.xhtml

during 2001 to 2009. This exclusion was done by calculating the PSA testing rate for the men in each practice for all 60 two-month periods within the observation period, and excluding a practice if in any two-month period all the following conditions were satisfied: (1) the testing rate was >3.5 SDs higher than the overall practice average; (2) more than 10 men were tested; (3) more than 5% of men not previously tested were tested in this period.

Statistical Analysis

The follow-up period for each man was calculated as the difference in years between registration start date (on or after the 1st January 2002) and censoring (defined above). The Kaplan-Meier failure function estimated the cumulative proportion of men exposed to at least one PSA test, and diagnosed with prostate cancer over the course of the 10-year period for all men. The log-rank test was then used to investigate relationships between characteristics of the men and risk of undergoing a PSA test. A Cox Proportional Hazards model and Wald test were also used to check that associations remained, with or without accounting for clustering by practice. For men with full ten-year follow-up and no diagnosis of prostate cancer (before or during follow-up), logistic and ordinal logistic regression were used to explore relationships between age group and the number of tests each man received.

The percentage of men retested within 365 days of their first test was explored by age category and PSA level for the CPRD data. For this analysis, all men in CPRD who had 365 days of follow-up post PSA test were included; as well as those diagnosed within the 365 days. Associations between age and retesting were investigated using logistic regression. Serum PSA levels for both the first and second test were used to determine the percentage of men diagnosed out of those who were retested, given their first and second PSA levels. Those known to have had a PSA test but no level recorded were assumed to have undetectable levels and therefore equivalent to zero.

Data on men who attended PSA screening as part of the ProtecT study[17] were used to explore how routine data on PSA tests compare with the tests carried out as part of a screening intervention. The ProtecT data were divided between men with LUTS (lower urinary tract symptoms) and no LUTS and compared with the CPRD dataset, by age group and further broken down by PSA level. LUTS was defined using 5 questions from the International Continence Society Male Short-Form (ICSmaleSF)

For peer review only - http://bmjopen.bmj.com/site/about/guidelines.xhtml

questionnaire.[21] Men were classified as having LUTS if any of the following were true: (1) urinating every two hours or more during the day; (2) urinating at least twice during night; or (3) suffering ‘sometimes’, ‘most of the time’ or ‘all of the time’ from delayed urination, rushing to urinate or leaking before reaching the toilet. Men diagnosed with prostate cancer before having their first PSA test in ProtecT were removed and, as with CPRD, those with a PSA test date but no level recorded were assumed to be undetectable and therefore equivalent to zero. PSA level was broken down into the following categories: PSA<3, 3≤PSA<4, 4≤PSA<6, 6≤PSA<10 and PSA≥10.

As an additional exploratory analysis, the percentage of men undergoing prostate biopsy and percentage diagnosed with prostate cancer within 365 days of their PSA test is also presented for men in the CPRD dataset and the two groups of ProtecT participants (LUTS and no LUTS). Comparisons between cohorts, and between risk groups within a cohort, were made using logistic and ordinal logistic regression. Within CPRD, biopsies and diagnoses were detected using medcodes provided by CPRD which correspond to Read-codes which are used in General Practice in the UK. Lists used are in the Supplementary material, Table S1.

For peer review only - http://bmjopen.bmj.com/site/about/guidelines.xhtml

RESULTS

Final cohort

In total, 450,000 men from 578 primary care practices across all regions of the UK (excluding London) were included in the CPRD data extract. Of these 450,000 men: 14 were removed due to missing or conflicting data; 303 were listed as having died before 2002; 2,184 had a diagnosis of prostate cancer date before 2002; and 369 patients had no follow up. From the remaining 447,130 patients, 12,894 (3%) men were removed as they were attendees of 19 practices suspected of participating in research involving practice-wide PSA testing. After the removal of these practices, the final sample was 434,236 men from 558 practices. Of these, 161,478 (37%) had the full 10-year follow up.

Risk of PSA testing and PCa diagnosis

The men were followed up for a cohort total of 2,963,645 person-years (median 8.25 years, IQR 3.83-10.00). Between 2002 and 2011 inclusively, 120,697 (28%) men received at least one PSA test and 7,538 (2%) men received a prostate cancer diagnosis. The cumulative 1, 5 and 10-year risks of receiving a PSA test were 5.1% (95% C.I. 5.0 to 5.2%), 21.4% (95% C.I. 21.3 to 21.5%) and 39.2% (95% C.I. 39.0 to 39.4%) respectively. The Kaplan-Meier curve illustrates the cumulative risk of PSA testing over the 10-year period, along with the age-specific cumulative risks (*Figure 1a*). A similar trend was seen in prostate cancer diagnoses (*Figure 1b*). The cumulative 1, 5 and 10 year risks of receiving a prostate cancer diagnosis were 0.2% (95% C.I. 0.2 to 0.2%), 1.0% (95% C.I. 1.0 to 1.1%) and 2.7% (95% C.I. 2.7 to 2.8%) respectively.

Table 1 shows the risks by age group, region, IMD quartiles and testing history. The risk of receiving a PSA test for men in the lowest age category (45-49 years) was substantially lower than the highest age category (65-69 years), with 10-year risks of exposure to PSA testing of 25.2% and 53.0% respectively ($P<0.001$). Likewise, the risk of diagnosis was also lower, with 10-year risks of 0.5% and 6.3% for age groups (45-49 years) and (65-69 years) respectively.

For peer review only - http://bmjopen.bmj.com/site/about/guidelines.xhtml

Table 1. Factors that influence the risk of having a PSA test/PCa diagnosis

		PSA testing			PCa diagnosis		
	N (%)	Men who had at least 1 PSA test	10 year risk % (95% C.I)£	P value	Men who had a PCa diagnosis	10 year risk % (95% C.I)£	P value
All men	434,236 (100%)	120,697	39.19 (39.01, 39.38)		7,538	2.72 (2.66, 2.78)	
**Age (in 2002)**							
45-49	104,782 (24%)	17,297	25.20 (24.86, 25.55)		296	0.49 (0.44, 0.55)	
50-54	100,211 (23%)	24,162	34.70 (34.33, 35.08)		858	1.40 (1.31, 1.50)	
55-59	97,224 (22%)	30,328	43.08 (42.69, 43.47)	p<0.001*	1,700	2.76 (2.63, 2.90)	p<0.001*
60-64	71,637 (17%)	25,518	48.57 (48.11, 49.04)		2,179	4.67 (4.47, 4.87)	
65-69	60,381 (14%)	23,392	52.95 (52.44, 53.45)		2,505	6.28 (6.04, 6.53)	
**Region**							
South East Coast	51,494 (12%)	17,434	47.45 (46.90, 48.01)		998	3.14 (2.95,3.34)	
Wales	35,277 (8%)	12,119	45.02 (44.40, 45.66)		689	2.79 (2.59, 3.01)	
Northern Ireland	12,730 (3%)	4,515	43.69 (42.70, 44.69)		264	2.75 (2.44, 3.11)	
South Central	53,577 (12%)	16,383	42.45 (41.93, 42.98)		976	2.79 (2.62, 2.98)	
South West	44,060 (10%)	12,399	40.82 (40.22, 41.42)		777	2.96 (2.75, 3.18)	
West Midlands	40,677 (9%)	11,453	39.28 (38.69, 39.88)	p<0.001§	704	2.66 (2.47, 2.86)	p<0.001§
North West	56,484 (13%)	16,340	38.88 (38.39, 39.37)		994	2.54 (2.38, 2.70)	
East of England	47,851 (11%)	12,386	38.85 (38.26, 39.44)		810	2.88 (2.68, 3.09)	
Yorkshire & the Humber	18,717 (4%)	4,131	35.49 (34.51, 36.50)		251	2.40 (2.10, 2.75)	
East Midlands	19,539 (5%)	4,466	34.43 (33.50, 35.38)		260	2.40 (2.10, 2.75)	
North East	8,113 (2%)	1,859	30.49 (29.31, 31.71)		123	2.25 (1.88, 2.69)	
Scotland	45,717 (11%)	7,212	23.82 (23.31, 24.33)		692	2.39 (2.21, 2.58)	
**Index of Multiple Deprivation (quartiles)**							
1-5 (Least deprived)	84,706 (20%)	29,422	46.26 (45.84, 46.67)		1,824	3.20 (3.06, 3.36)	
6-10	69,496 (16%)	21,611	42.45 (42.00, 42.91)		1,332	2.92 (2.76, 3.08)	
11-15	56,865 (13%)	14,596	36.32 (35.82, 36.82)	p<0.001*	916	2.54 (2.38, 2.72)	p<0.001*
16-20 (Most deprived)	40,833 (9%)	8,735	31.92 (31.33, 32.51)		483	1.92 (1.75, 2.10)	
No IMD recorded	182,336 (42%)	46,333	36.87 (36.58, 37.16)		2,983	2.63 (2.53, 2.73)	
**Pre-registration PSA test**							
Previously tested	27,211 (6%)	15,368	73.21 (72.54, 73.89)	p<0.001§	1,089	5.94 (5.59, 6.31)	p<0.001§
Not previously tested	407,025 (94%)	105,329	36.97 (36.78, 37.16)		6,449	2.51 (2.45, 2.57)	

£Kaplan Meier failure function at 10 years, *P for trend, §P across categories

For peer review only - http://bmjopen.bmj.com/site/about/guidelines.xhtml

PSA testing and diagnosis risks varied by region (p<0.001). The risks of testing and diagnosis were higher in more southern areas, especially the South East Coast (47.5% and 3.1% respectively) and Wales (45.0% and 2.8%). The lowest risks were found in Scotland (23.8% and 2.4%) and the North East (30.5% and 2.3%). Those living in areas of greater deprivation had a lower risk of testing (46.3% vs. 31.9%) and diagnosis (3.2% vs. 1.9%); p for trend<0.001.

Those who had received a PSA test prior to registration were substantially more likely to receive a PSA test and diagnosis than those who had not (73.2 vs 37.0 and 5.9 vs. 2.5 respectively); p<0.001.

Number of PSA tests

There were 157,586 men with complete 10-year follow-up and no prostate cancer. Of these, 57,491 men (36%) underwent at least one PSA test. Older age group was strongly related to a greater number of tests over 10 years (p<0.001, Table 2).

PSA levels and retesting

Data on PSA levels in CPRD were incomplete, but the median first PSA result of those tested with a result (n=119,175) was 1.23ng/ml (IQR=0.70-2.60; *Figure 2*). Assuming that those with a PSA test date but missing level (n=1,522, 1%) were undetectable and therefore ≈0, the median PSA result would be 1.20ng/ml (IQR=0.70-2.60). Removing the lowest age category (45-49) increased the median PSA to 1.30ng/ml (0.70-2.81), n=103,400. For the ProtecT men, the median PSA result of those collected (n=58,542) was 0.99ng/ml (IQR=0.60, 1.70) which remained the same after making the same assumption for the 27 men with a missing PSA result.

Of those PSA tested with a full year's follow up after their test (aged 50-69), 17,757/90,252 (20%) had a second test within a year of their first (Table 3). Undergoing a second PSA test within a year of the first test was strongly associated with a higher PSA level at the first test (OR per PSA category higher 1.83, 95% C.I. 1.80, 1.85; p<0.001). Those men with a PSA<3ng/ml were more likely to be retested within a year if they were in an older age group (OR per age category older 1.04, 95% confidence interval 1.04, 1.04; p<0.001). This trend was reversed for those men with a PSA≥3ng/ml

For peer review only - http://bmjopen.bmj.com/site/about/guidelines.xhtml

Table 2. Number of PSA tests received by men with full 10 year follow up and no prostate cancer diagnosis

Number of tests	0	1	2	3	4	5	6	7	8	9	≥10
All men	100,095 (64%)	28,561 (18%)	12,196 (8%)	6,047 (4%)	3,449 (2%)	2,050 (1%)	1,379 (1%)	929 (1%)	734 (<1%)	539 (<1%)	1,607 (1%)
**Age (in 2002)**											
45-49	28,998 (77%)	5,651 (15%)	1,744 (5%)	647 (2%)	284 (1%)	162 (<1%)	81 (<1%)	37 (<1%)	23 (<1%)	20 (<1%)	47 (<1%)
50-54	25,299 (68%)	6,846 (18%)	2,549 (7%)	1,132 (3%)	606 (2%)	324 (1%)	195 (1%)	107 (<1%)	100 (<1%)	63 (<1%)	150 (<1%)
55-59	21,471 (59%)	7,092 (20%)	3,176 (9%)	1,654 (5%)	866 (2%)	549 (2%)	384 (1%)	246 (1%)	195 (1%)	144 (<1%)	380 (1%)
60-64	13,903 (54%)	4,941 (19%)	2,508 (10%)	1,371 (5%)	917 (4%)	524 (2%)	354 (1%)	277 (1%)	212 (1%)	160 (1%)	482 (2%)
65-69	10,424 (50%)	4,031 (19%)	2,219 (11%)	1,243 (6%)	776 (4%)	491 (2%)	365 (2%)	262 (1%)	204 (1%)	152 (1%)	548 (3%)

For peer review only

For peer review only - http://bmjopen.bmj.com/site/about/guidelines.xhtml

Table 3. PSA levels* by age group in the CPRD data and Protect men (LUTS vs. no LUTS)

	Age group 50-54			Age group 55-59			Age group 60-64			Age group 65-69		
PSA level	CPRD$	LUTS£	No LUTS£	CPRD$	LUTS£	No LUTS£	CPRD$	LUTS£	No LUTS£	CPRD$	LUTS£	No LUTS£
**Number of men tested (%)**												
PSA<3~	17898 (87%)	5228 (95%)	10178 (96%)	21183 (80%)	5716 (90%)	10331 (92%)	16306 (73%)	4730 (83%)	7560 (86%)	13622 (66%)	3646 (78%)	4681 (82%)
3≤PSA<4	936 (5%)	138 (3%)	216 (2%)	1714 (6%)	276 (4%)	410 (4%)	1734 (8%)	412 (7%)	469 (5%)	1776 (9%)	393 (8%)	394 (7%)
4≤PSA<6	838 (4%)	79 (1%)	137 (1%)	1655 (6%)	201 (3%)	290 (3%)	1868 (8%)	262 (5%)	407 (5%)	2026 (10%)	331 (7%)	340 (6%)
6≤PSA<10	500 (2%)	29 (1%)	61 (1%)	1092 (4%)	98 (2%)	145 (1%)	1340 (6%)	174 (3%)	193 (2%)	1642 (8%)	201 (4%)	188 (3%)
10≤PSA<20	294 (1%)	13 (<1%)	16 (<1%)	541 (2%)	29 (<1%)	41 (<1%)	717 (3%)	65 (1%)	96 (1%)	925 (4%)	88 (2%)	77 (1%)
PSA≥20	165 (1%)	3 (<1%)	7 (<1%)	350 (1%)	15 (<1%)	21 (<1%)	513 (2%)	28 (<1%)	39 (<1%)	614 (3%)	38 (1%)	52 (<1%)
**Number retested within 1 year of their first PSA test (%)**												
PSA<3~	1707 (10%)			2391 (11%)			2294 (14%)			2130 (16%)		
3≤PSA<4	256 (27%)			336 (20%)			308 (18%)			359 20%)		
4≤PSA<6	507 (61%)			902 (55%)			927 (50%)			819 (40%)		
6≤PSA<10	336 (67%)			681 (62%)			778 (58%)			934 (57%)		
10≤PSA<20	185 (63%)			315 (58%)			408 (57%)			489 (53%)		
PSA≥20	82 (49%)			163 (47%)			215 (42%)			235 (38%)		
**Number biopsied within 1 year of their first PSA test (%)**												
PSA<3~	20 (<1%)	0 (0%)	0 (0%)	36 (<1%)	0 (0%)	0 (0%)	28 (<1%)	0 (0%)	0 (0%)	28 (<1%)	0 (0%)	1 (<1%)
3≤PSA<4	13 (1%)	122 (88%)	174 (80%)	7 (<1%)	245 88%)	347 (83%)	10 (1%)	345 (84%)	374 (81%)	5 (<1%)	316 (81%)	307 (78%)
4≤PSA<6	61 (7%)	74 (94%)	122 (88%)	92 (6%)	186 (92%)	254 (86%)	87 (5%)	240 (90%)	339 (84%)	50 (3%)	278 (85%)	274 (81%)
6≤PSA<10	51 (10%)	27 (93%)	56 (92%)	133 (12%)	92 (91%)	134 (92%)	128 (10%)	160 (93%)	166 (86%)	139 (9%)	168 (84%)	161 (87%)
10≤PSA<20	36 (12%)	13 (93%)	16 (100%)	84 (16%)	26 (90%)	41 (98%)	109 (15%)	60 (94%)	89 (92%)	116 (13%)	81 (92%)	65 (87%)
PSA≥20	18 (11%)	3 (100%)	5 (71%)	56 (16%)	11 (69%)	17 (81%)	62 (12%)	23 (82%)	33 (87%)	66 (11%)	30 (81%)	39 (75%)
**Number diagnosed within 1 year of their first PSA test - with or without biopsy (%)**												
PSA<3~	18 (<1%)	0 (0%)	2 (<1%)	52 (<1%)	1 (<1%)	2 (<1%)	55 (<1%)	2 (<1%)	1 (<1%)	64 (<1%)	2 (<1%)	1 (<1%)
3≤PSA<4	19 (2%)	28 (20%)	47 (22%)	25 (2%)	62 (22%)	102 (24%)	18 (1%)	98 (24%)	115 (25%)	10 (1%)	87 (22%)	111 (28%)
4≤PSA<6	80 (10%)	29 (37%)	44 (32%)	124 (8%)	64 (32%)	95 (32%)	129 (7%)	80 (30%)	139 (34%)	79 (4%)	90 (28%)	119 (35%)
6≤PSA<10	99 (20%)	15 (52%)	27 (44%)	186 (17%)	37 (37%)	66 (45%)	258 (19%)	70 (41%)	96 (49%)	264 (16%)	74 (37%)	84 (45%)
10≤PSA<20	87 (30%)	6 (43%)	12 (75%)	155 (29%)	18 (62%)	29 (69%)	242 (34%)	35 (55%)	76 (78%)	296 (32%)	49 (56%)	47 (63%)
PSA≥20	86 (51%)	3 (100%)	7 (100%)	220 (63%)	11 (69%)	16 (76%)	347 (68%)	23 (82%)	34 (89%)	421 (69%)	32 (86%)	42 (81%)

*Those without a test date could not be included as we could not determine whether they had a full years follow up post-test, ~Those with a PSA test but missing PSA level were assumed to have a score that was undetectable and therefore below 3, $Data taken between Jan 2002-Dec 2011 for PSA tests taken in Jan 2002-Dec 2010 - any men without 1 full years follow up post-test were removed, £Data taken from the ProtecT study [17] between Jan 2002-Jan 2010 for PSA tests taken from Jan 2002-Jan 2009

For peer review only

For peer review only - http://bmjopen.bmj.com/site/about/guidelines.xhtml

where those in an older age group were less likely to be retested within a year than those in a younger age group (OR per age category older 0.98, 95% C.I. 0.97, 0.98; $p<0.001$).

Older men were also at greater risk of having a higher PSA test than those in younger age categories, for ProtecT and CPRD data, (for the CPRD cohort, OR per age group older 1.08, 95% C.I. 1.07, 1.08; $p<0.001$). On average, those ProtecT participants presenting with LUTS appeared to have higher PSA levels than those with no LUTS, while men in CPRD had the highest PSA results (*Table 3).*

Subsequent biopsies and diagnoses

From the ProtecT data, 22,200 men were identified as having LUTS (based on our definition) at the consultation for their PSA test and 36,364 men did not have LUTS. For men with a PSA level of 3ng/ml or higher, biopsy and diagnosis rates were much higher in ProtecT participants than CPRD. This remained true, even when those with a high PSA level were confirmed high in a further test (*Table S2*). Furthermore, for men in the CPRD cohort, a lower proportion underwent biopsy than were subsequently diagnosed. Overall, the odds of diagnosis within a year of a PSA test was 3 times higher in the ProtecT study compared with the CPRD data for those with PSA≥3, (OR 2.99, 95% C.I. 2.80, 3.18; $p<0.001$).

For CPRD, as expected, men with higher PSA level were more likely to be diagnosed (OR per PSA category higher 3.46, 95% C.I. 3.37, 3.56; $p<0.001$), as were older men (OR per age category higher 1.08, 95% C.I. 1.07, 1.08; $p<0.001$). For those aged between 50 and 69, the biopsy rates were <1%, 1%, 5%, 10% and 13% for PSA categories PSA<3, 3≤PSA<4, 4≤PSA<6, 6≤PSA<10 and PSA≥10 respectively. The diagnosis rates were <1%, 1%, 6%, 18% and 59% respectively.

For peer review only - http://bmjopen.bmj.com/site/about/guidelines.xhtml

DISCUSSION

This paper has examined the risk of receiving a PSA test over a 10-year period in a large retrospective cohort of men aged 45-69 in the UK (excluding London). The 10-year risk of undergoing a PSA test was estimated at 39.2% while the 10-year risk of receiving a prostate cancer diagnosis was estimated at 2.7%. Higher rates of both testing and diagnoses were associated with older age, more southerly region of residence, less deprived index of multiple deprivation (IMD), and a history of PSA testing. For all age groups and PSA levels, the proportion of men undergoing biopsy, and subsequently diagnosed with prostate cancer following a PSA test in UK general practice is low when compared to men in the PSA testing programme undertaken as part of the ProtecT trial.[17]

Overall the number of men without a prior diagnosis of prostate cancer receiving at least one PSA test over 10-years is high, especially given the lack of a screening programme in the UK. The proportion of men undergoing their first test in the follow-up period increases steadily throughout the ten-year period. The higher rates of testing in older men is consistent with the findings of other studies of UK general practice[8-10] although not with the age-distribution of men agreeing to participate in the ProtecT study, the latter being in close agreement with the male population age distribution, with the majority of men being younger than 60 years.[17] These findings suggest that interest in prostate cancer screening is not concentrated in the older age groups, and that the greater incidence of testing in older men is likely to arise due to other diagnostic indications for the PSA test. The increase in testing with age could be due to an increase in lower urinary tracts symptoms with age[22] or with the GP wanting to rule out the possibly of prostate cancer[23] despite this rarely being the cause of such symptoms.[21 24] It is also thought that the PSA level is a useful indicator of prostate volume and may inform the choice between treatment options for BPH and other benign conditions.[12 25]

The observation of greater testing of men living in more affluent areas is consistent with previous studies.[8-10] This association presumably arises from more affluent men being more likely to request a test, or through general practices serving more affluent areas being more likely to promote the test to their male patients. There is some evidence to suggest that prostate cancer is more prevalent in areas of lower deprivation;[26] however, the extent to which PSA testing patterns inform this is difficult to determine.

For peer review only - http://bmjopen.bmj.com/site/about/guidelines.xhtml

Overall, 11% of men with 10 years of follow-up were tested three or more times. This varied by age group, 20% of men aged 65 to 69 years at the outset being tested three or more times, compared to 3% of men aged 45 to 49 years. Two major trials of prostate cancer screening have employed repeated PSA testing,[27 28] although the association with age would not be expected if the programmes in those trials were being followed by UK general practitioners, and certainly not a greater number of older men undergoing multiple tests.

ProtecT participants with LUTS had slightly higher PSA levels on average than those without LUTS. Whilst 78% of men in UK general practice undergoing a PSA test were found to have a level below 3ng/ml, on average PSA levels were higher than seen in the ProtecT study. In part this will be due to the older age profile of men in UK general practice compared to ProtecT participants, but it is also consistent with more tests in General Practice being undertaken to inform a diagnosis of LUTS, as LUTS are associated with elevated PSA levels (there is no reason to suppose a higher prevalence of non-symptomatic and undiagnosed prostate cancer in CPRD and ProtecT general practices). A strong association was observed for all age groups between higher PSA levels at a first test and the probability of a man undergoing a second test within one year, indicating that the results of the PSA tests did inform clinical management.

The incidence of biopsy and prostate cancer diagnosis in the CPRD cohort suggests that a PSA of 4ng/ml or more was being used in UK general practice as a trigger for further diagnostic investigations. The incidence of biopsy in the CPRD cohort is very low, and the fact that there are fewer biopsies than prostate cancer diagnoses suggests that either many more men were refusing biopsy, perhaps due to the full screening process not being discussed at the time of PSA testing, or that biopsy is being under-recorded in general practice data. However, even allowing for a degree of under-reporting, only a small minority of men with high PSA levels were recorded as having had a biopsy, which contrasts with 80% plus of ProtecT men with PSA of 3ng/ml or higher undergoing the investigation. Furthermore, the risk of a prostate cancer diagnosis in the CPRD cohort is much lower than in comparable men participating in the ProtecT study prospective PSA testing programme. These findings are again consistent with the majority of PSA tests in UK general practice being undertaken

For peer review only - http://bmjopen.bmj.com/site/about/guidelines.xhtml

For peer review only

to inform the diagnosis and management of LUTS in older men, with no intention of screening for prostate cancer.

Strengths and Limitations

The major strength of this investigation is the use of CPRD data which has allowed a large retrospective cohort of men to be constructed, and followed-up for a period of up to ten years. The use of CPRD data is also behind the key weakness of this study: the completeness of some data items is uncertain, with the recorded diagnoses outnumbering the recorded biopsies indicating that the latter are under-recorded, presumably even when cancer is diagnosed.

We did not attempt to distinguish those tests undertaken in men presenting with and without symptoms, and this could be considered a further limitation of our study. Screening aims to diagnose a disease before symptoms arise. However, prostate cancer rarely results in LUTS and sexual symptoms until it is at an advanced stage. For the vast majority of men with urinary and sexual symptoms the cause is benign, and in fact men with an elevated PSA are less likely to be diagnosed with prostate cancer if they also have LUTS or impaired sexual function.[21 29] The pattern of PSA testing in the CPRD cohort suggests that many PSA tests are being undertaken to inform the diagnosis and management of LUTS, and knowing which men had been PSA tested because of a presentation with symptoms would have lent further support to this hypothesis.

For peer review only - http://bmjopen.bmj.com/site/about/guidelines.xhtml

CONCLUSION

In UK general practice, 39.2% of men aged 45 to 69 years and initially free of prostate cancer undergo at least one PSA test during a 10-year follow-up period (2002 to 2011). However, testing rates are higher in the older age groups, and high PSA levels are commonly not followed up by a biopsy, required for the diagnosis of prostate cancer. Hence it is likely that a high proportion of these tests are related to investigations or management of lower urinary tract symptoms and other benign conditions, and cannot be considered as part of an effective (informal) effort to screen for prostate cancer.

For peer review only

AUTHOR CONTRIBUTIONS

ELT, SEO, YBS, JAL, DEN, FCH, JLD, RMM and CM contributed to the design of the study and formulated the research question. SH, ELT, EIW, RMM and CM contributed to the acquisition of the data. GJY, SH, ELT, EIW, SE, RMM and CM made substantial contributions to the analysis and interpretation of data for the work. GJY, SH and CM wrote the first draft. All authors commented on the drafts and revised it critically for important intellectual content. All authors approved the final manuscript and are accountable for the accuracy and integrity of the work.

ACKNOWLEDGEMENTS

We acknowledge the contribution of the CAP trial group. Investigators: RMM (Lead PI), JLD (PI), DEN (PI), FCH (PI), ELT (trial Co-ordinator), CM (statistician), Jonathan Sterne (statistician), Sian Noble (health economist), JAL. Research staff: Elizabeth Hill, Siaw Yien Ng, Naomi Williams, Liz Down (data manager), EIW (data manager), GJY (statistician), Joanna Thorn (health economist), Charlotte Davies, Laura Hughes, Mari-Anne Rowlands and Lindsey Bell. Management committee: ELT (Chair), RMM, JLD, CM, Jonathan Sterne, Sian Noble, YBS, JAL, SEO, Peter Brindle and SE. Trial steering committee: Michael Baum (Chair), Peter Albertsen, Tracy Roberts, Mary Robinson, Jan Adolfsson, David Dearnaley, AZ, Fritz Schröder, Tim Peters, Peter Holding, Teresa Lennon, Sue Bonnington, Malcolm Mason, Jon Oxley, RMM, JLD, DEN, FCH, ELT and JAL. Data monitoring committee: Lars Holmberg (Chair), Robert Pickard, Simon Thompson and Usha Menon. Cause of Death Committee: Peter Albertsen (Chair), Colette Reid, John McFarlane, Jon Oxley, Mary Robinson, Jan Adolfsson, Michael Baum, Anthony Zietman, Amit Bahl and Anthony Koupparis. Administrative staff: Marta Tazewell and Genevieve Hatton-Brown. We wish to extend our thanks to Pete Shiarly for the development of bespoke databases. We also wish to acknowledge the contribution of all members of the ProtecT study research groups. Thanks are extended to the Cancer Registries and staff at the Health and Social Care Information Centre. We acknowledge the contributions of the ProtecT and CAP study participants, investigators, researchers, data monitoring committees, and trial steering committees.

For peer review only

SUPPORT AND FUNDING

This work was supported by Cancer Research UK and the UK Department of Health for the CAP trial (C11043/A4286, C18281/A8145, C18281/A11326, and C18281/A15064). CAP is sponsored by the University of Bristol and is registered at Current Controlled Trials (ISRCTN92187251). The ProtecT trial is funded by the UK National Institute for Health Research (NIHR), Health Technology Assessment Programme (projects 96/20/06, 96/20/99). The views and opinions expressed herein are our own and do not necessarily reflect those of the Department of Health. The ProtecT trial is sponsored by the University of Oxford and is registered at Current Controlled Trials (ISRCTN20141297) and Clinical Trials.Gov (NCT02044172).

We acknowledge the support from the Oxford NIHR Biomedical Research Centre through the Surgical Innovation and Evaluation Theme, and the Surgical Interventional Trials Unit, and Cancer Research UK through the Oxford Cancer Research Centre.

SH is a Wellcome Trust-funded PhD student with Grant Code 102432/Z/13/Z. JLD is supported, in part, by the NIHR Collaboration for Leadership in Applied Health Research and Care West, hosted by University Hospitals Bristol NHS Foundation Trust. FCH is supported, in part, by the Oxford NIHR Biomedical Research Centre and the Cancer Research UK Oxford Centre. RMM is supported, in part, by University Hospitals Bristol NHS Foundation Trust National Institute for Health Research Bristol Nutrition Biomedical Research Unit and CRUK (C18281/A19169). JLD, FCH and DEN are NIHR Senior Investigators. The views and opinions expressed herein are the authors own and do not necessarily reflect those of the Department of Health. The funders and sponsor had no role in the design and conduct of the study, or the preparation of the report or decision to publish. The corresponding author had full access to the data and takes responsibility for the decision to submit for publication.

CONFLICT OF INTEREST

The authors declare no conflict of interest.

For peer review only

DATA SHARING

The authors do not have permission to share data obtained from the Clinical Practice Research Datalink (CPRD). Baseline data from the ProtecT trial may be available on application (email info-protect@bris.ac.uk).

For peer review only

For peer review only - http://bmjopen.bmj.com/site/about/guidelines.xhtml

REFERENCES

1. Harvey CJ, Pilcher J, Richenberg J, et al. Applications of transrectal ultrasound in prostate cancer. *Br J Radiol* 2012;85 Spec No 1:S3-17. doi: 10.1259/bjr/56357549
2. Prensner JR, Rubin MA, Wei JT, et al. Beyond PSA: The Next Generation of Prostate Cancer Biomarkers. *Science Translational Medicine* 2012;4(127) doi: ARTN 127rv310.1126/scitranslmed.3003180
3. Schroder FH, Hugosson J, Roobol MJ, et al. Screening and prostate cancer mortality: results of the European Randomised Study of Screening for Prostate Cancer (ERSPC) at 13 years of follow-up. *Lancet* 2014;384(9959):2027-35. doi: 10.1016/S0140-6736(14)60525-0
4. Chou R, Croswell JM, Dana T, et al. Screening for Prostate Cancer: A Review of the Evidence for the US Preventive Services Task Force. *Ann Intern Med* 2011;155(11):762-U94. doi: 10.7326/0003-4819-155-11-201112060-00375
5. Gulati R, Mariotto AB, Chen S, et al. Long-term projections of the harm-benefit trade-off in prostate cancer screening are more favorable than previous short-term estimates. *J Clin Epidemiol* 2011;64(12):1412-17. doi: 10.1016/j.jclinepi.2011.06.011
6. Donovan JL, Hamdy FC, Lane JA, et al. Patient-Reported Outcomes after Monitoring, Surgery, or Radiotherapy for Prostate Cancer. *New Engl J Med* 2016;375(15):1425-37. doi: 10.1056/NEJMoa1606221
7. Pickles K, Carter SM, Rychetnik L. Doctors' approaches to PSA testing and overdiagnosis in primary healthcare: a qualitative study. *BMJ Open* 2015;5(3) doi: ARTN e00636710.1136/bmjopen-2014-006367
8. Melia J, Moss S, Johns L. Rates of prostate-specific antigen testing in general practice in England and Wales in asymptomatic and symptomatic patients: a cross-sectional study. *BJU International* 2004;94(1):51-56. doi: 10.1111/j.1464-410X.2004.04832.x
9. Williams N, Hughes LJ, Turner EL, et al. Prostate-specific antigen testing rates remain low in UK general practice: a cross-sectional study in six English cities. *BJU International* 2011;108(9):1402-08. doi: 10.1111/j.1464-410X.2011.10163.x
10. Moss S, Melia J, Sutton J, et al. Prostate-specific antigen testing rates and referral patterns from general practice data in England. *Int J Clin Pract* 2016;70(4):312-18. doi: 10.1111/ijcp.12784
11. Nderitu P, Van Hemelrijck M, Ashworth M, et al. Prostate-specific antigen testing in inner London general practices: are those at higher risk most likely to get tested? *BMJ Open* 2016;6(7) doi: ARTN e01135610.1136/bmjopen-2016-011356
12. Roehrborn CG, McConnell JD, Lieber M, et al. Serum prostate-specific antigen concentration is a powerful predictor of acute urinary retention and need for surgery in men with clinical benign prostatic hyperplasia. *Urology* 1999;53(3):473-80. doi: Doi 10.1016/S0090-4295(98)00654-2
13. National Institute of Health and Care Excellence [NICE]. LUTS in men Available at: https://cks.nice.org.uk/luts-in-men#!topicsummary (Accessed 14 March 2017)2015 [Available from: https://cks.nice.org.uk/luts-in-men#!topicsummary accessed 14/03/2017.
14. National Institute for Health and Care Excellence [NICE]. Lower urinary tract symptoms in men: management Available at: https://www.nice.org.uk/guidance/cg97/chapter/1-Recommendations (Accessed 14 March 2017)2010 [Available from: Available at: https://www.nice.org.uk/guidance/cg97/chapter/1-Recommendations (Accessed 14 March 2017) accessed 14/03/2017.
15. Nordstrom T, Aly M, Clements MS, et al. Prostate-specific Antigen (PSA) Testing Is Prevalent and Increasing in Stockholm County, Sweden, Despite No Recommendations for PSA Screening: Results from a Population-based Study, 2003-2011. *European Urology* 2013;63(3):419-25. doi: 10.1016/j.eururo.2012.10.001
16. Hamoen EHJ, Reukers DFM, Numans ME, et al. Discrepancies between guidelines and clinical practice regarding prostate-specific antigen testing. *Family Practice* 2013;30(6):648-54. doi: 10.1093/fampra/cmt045
17. Lane JA, Donovan JL, Davis M, et al. Active monitoring, radical prostatectomy, or radiotherapy for localised prostate cancer: study design and diagnostic and baseline results of the ProtecT randomised phase 3 trial. *Lancet Oncology* 2014;15(10):1109-18. doi: 10.1016/S1470-2045(14)70361-4

For peer review only - http://bmjopen.bmj.com/site/about/guidelines.xhtml

18. Walley T, Mantgani A. The UK General Practice Research Database. *Lancet* 1997;350(9084):1097-99. doi: Doi 10.1016/S0140-6736(97)04248-7
19. Herrett E, Gallagher AM, Bhaskaran K, et al. Data Resource Profile: Clinical Practice Research Datalink (CPRD). *International Journal of Epidemiology* 2015;44(3):827-36. doi: 10.1093/ije/dyv098
20. Campbell J, Dedman DJ, Eaton SC, et al. Is the CPRD GOLD Population Comparable to the UK Population? *Pharmacoepidemiology and Drug Safety* 2013;22:280-81.
21. Collin S, Metcalfe C, Donovan J, et al. Associations of lower urinary tract symptoms with prostate-specific antigen levels and screen-detected localized and advanced prostate cancer: A case-control study nested within the UK population-based protect (prostate testing for cancer and treatment) study. *Neurourology and Urodynamics* 2008;27(7):583-84.
22. Andersson SO, Rashidkhani B, Karlberg L, et al. Prevalence of lower urinary tract symptoms in men aged 45-79 years: a population-based study of 40,000 Swedish men. *BJU International* 2004;94(3):327-31. doi: 10.1111/j.1464-410X.2004.04930.x
23. Young JM, Muscatello DJ, Ward JE. Are men with lower urinary tract symptoms at increased risk of prostate cancer? A systematic review and critique of the available evidence. *BJU International* 2000;85(9):1037-48. doi: DOI 10.1046/j.1464-410x.2000.00659.x
24. Martin RM, Vatten L, Gunnell D, et al. Lower urinary tract symptoms and risk of prostate cancer: The HUNT 2 Cohort, Norway. *International Journal of Cancer* 2008;123(8):1924-28. doi: 10.1002/ijc.23713
25. Speakman MJ, Kirby RS, Joyce A, et al. Guideline for the primary care management of male lower urinary tract symptoms. *BJU International* 2004;93(7):985-90. doi: 10.1111/j.1464-410X.2004.04765.x
26. National Cancer Registration and Analysis Service. Cancer by Deprivation in England 1996 - 2011 Available at: http://www.ncin.org.uk/about_ncin/cancer_by_deprivation_in_england (Accessed 14 March 2017)2014 [Available from: http://www.ncin.org.uk/about_ncin/cancer_by_deprivation_in_england.
27. Schroeder FH, Hugosson J, Roobol MJ, et al. Screening and Prostate-Cancer Mortality in a Randomized European Study. *New Engl J Med* 2009;360(13):1320-28. doi: 10.1056/NEJMoa0810084
28. Andriole GL, Grubb RL, Buys SS, et al. Mortality Results from a Randomized Prostate-Cancer Screening Trial. *New Engl J Med* 2009;360(13):1310-19. doi: 10.1056/NEJMoa0810696
29. Collin SM, Metcalfe C, Donovan J, et al. Associations of Sexual Dysfunction with Localized and Advanced Prostate Cancer: A Case-Control Study Nested within the Uk Population-Based Protect (Prostate Testing for Cancer and Treatment) Study. *Eur Urol Suppl* 2009;8(4):139-39.

For peer review only

For peer review only - http://bmjopen.bmj.com/site/about/guidelines.xhtml

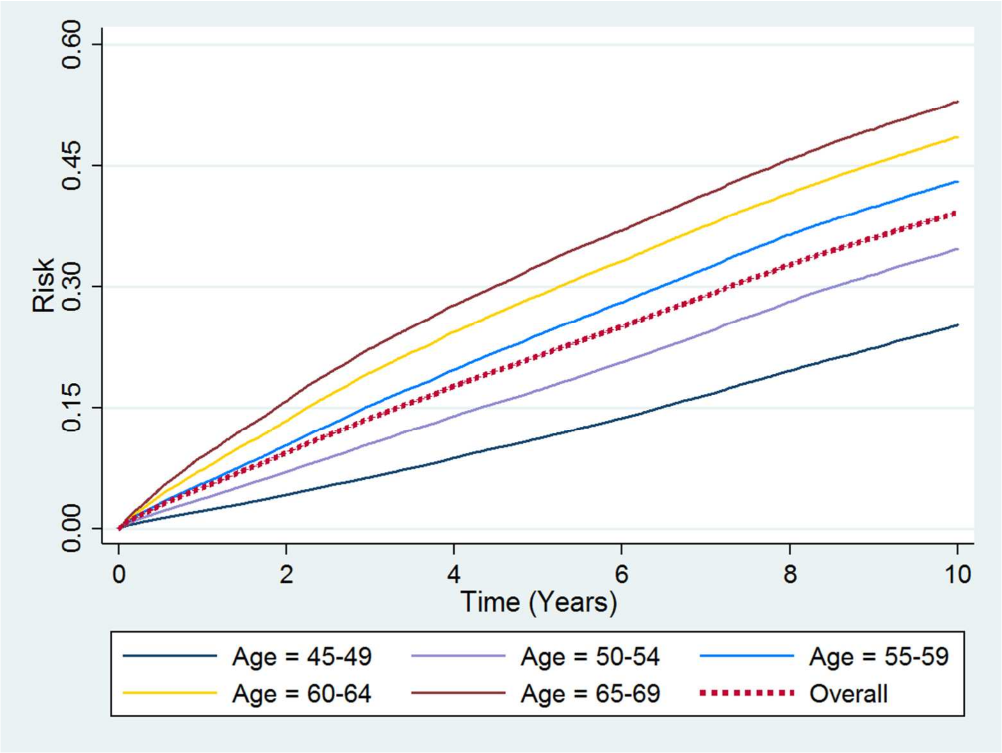

Figure 1a. Kaplan Meier failure estimate: Cumulative risk over 10 years of receiving a PSA test, by age group, between 2002 & 2012

101x76mm (300 x 300 DPI)

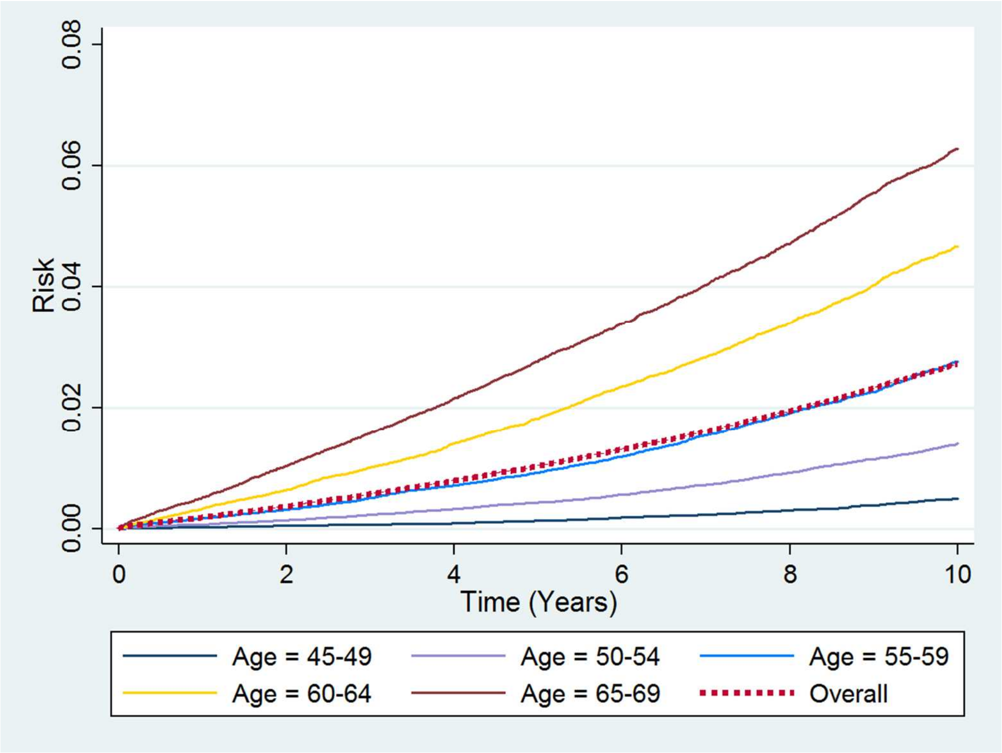

Figure 1b. Kaplan Meier failure estimate: Cumulative risk over 10 years of receiving a PCa diagnosis, by age group, between 2002 & 2012

101x76mm (300 x 300 DPI)

For peer review only - http://bmjopen.bmj.com/site/about/guidelines.xhtml

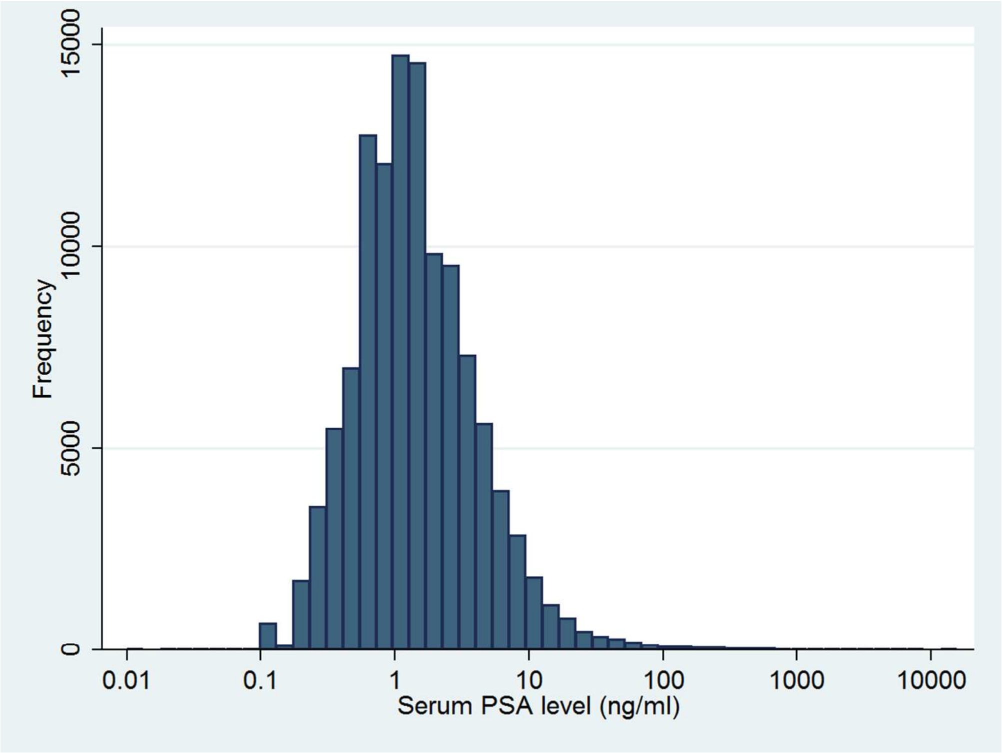

Figure 2. Distribution of PSA levels on the log scale

101x76mm (300 x 300 DPI)

For peer review only - http://bmjopen.bmj.com/site/about/guidelines.xhtml

Supplementary material

Table S1. List of used medical codes for prostate cancer biopsies and diagnoses

Biopsies		Diagnoses	
Medcode	Readterm	Medcode	Readterm
1069	*Transrectal needle biopsy of prostate*	780	*Malignant neoplasm of prostate*
7908	*Open biopsy of prostate*	6328	*Carcinoma in situ of prostate*
7909	*Unspec diagnostic cystoscopic exam bladder & biopsy prostate*	10178	*Gleason grading of prostate*
12391	*Transurethral biopsy prostate*	18503	*Gleason prostate grade 2-4 (low)*
22297	*Trucut transperineal biopsy of prostate*	18612	*Gleason prostate grade 5-7 (medium)*
22473	*Transperineal needle biopsy of prostate*	26081	*Gleason prostate grade 8-10 (high)*
22719	*Endoscopic punch biopsy of prostate*	37306	*Personal history of malignant neoplasm of prostate*
		102314	*History of prostate cancer*

Table S2. The percentage of men diagnosed with prostate cancer in CPRD based on their first and second PSA test

**% diagnosed (n/N)**	**Second test (within 1 year) PSA level**					
	No 2nd test	PSA<3˜	PSA<3*	3≤PSA<6	6≤PSA<10	PSA≥10
**First test PSA level**						
PSA<3*	<1% (119/60487)	<1% (35/7692)	<1% (37/7876)	1% (6/477)	18% (17/97)	14% (10/72)
3≤PSA<6	3% (234/8133)	1% (6/748)	2% (14/850)	5% (162/3027)	14% (66/483)	15% (8/54)
6≤PSA<10	24% (452/1845)	1% (3/230)	6% (18/302)	4% (23/622)	17% (269/1556)	18% (45/249)
PSA≥10	61% (1232/2030)	6% (11/183)	14% (35/246)	3% (6/227)	9% (29/338)	43% (552/1281)

*PSA tests without a level recorded were assumed to be below the detection level and hence <3ng/ml
˜Men with second PSA tests without a level recorded are not included

For peer review only

BMJ Open

Prostate Specific Antigen (PSA) testing of men in UK general practice: a 10-year longitudinal cohort study

Journal:	*BMJ Open*
Manuscript ID	bmjopen-2017-017729.R1
Article Type:	Research
Date Submitted by the Author:	12-Jul-2017
Complete List of Authors:	Young, Grace; University of Bristol, School of Social and Community Medicine; Bristol Randomised Trials Collaboration, University of Bristol<br>Harrison, Sean; University of Bristol School of Social and Community Medicine<br>Turner, Emma; University of Bristol, School of Social and Community Medicine<br>Walsh, Eleanor; University of Bristol, School of Social and Community Medicine<br>Oliver, Steven; University of York, The Hull York Medical School<br>Ben-Shlomo, Yoav; University of Bristol,<br>Evans, Simon; Royal United Hospital, Urology Department<br>Lane, Athene; University of Bristol, School of Social and Community Medicine; Bristol Randomised Trials Collaboration, University of Bristol<br>Neal, David; University of Oxford Nuffield Department of Surgical Sciences<br>Hamdy, Freddie; University of Oxford Nuffield Department of Surgical Sciences<br>Donovan, Jenny; University of Bristol School of Social and Community Medicine<br>Martin, Richard; University of Bristol, Social Medicine<br>Metcalfe, Chris; University of Bristol, Social Medicine; Bristol Randomised Trials Collaboration, University of Bristol
**Primary Subject Heading**:	Public health
Secondary Subject Heading:	Diagnostics, Epidemiology, Urology
Keywords:	PSA testing, screening, primary health care, prostate cancer

SCHOLARONE™
Manuscripts

Prostate Specific Antigen (PSA) testing of men in UK general practice: a 10-year longitudinal cohort study

Running title: PSA testing in UK general practice: 2002-2012

Ms Grace J Young[1,2]* MSc, Mr Sean Harrison[1]* MA (Cantab), Dr Emma L Turner[1] PhD, Ms Eleanor I Walsh[1] MSc, Dr Steven E Oliver[3] PhD, Prof Yoav Ben-Shlomo[1] PhD, Mr Simon Evans[4] MRCS, Dr J Athene Lane[1,2] PhD, Prof David E Neal[5] FMedSci, Prof Freddie C Hamdy[5] FMedSci, Prof Jenny L Donovan[1] PhD, Prof Richard M Martin[1] PhD, Dr Chris Metcalfe[1,2] PhD

*denoting joint first authorship

[1]Address: School of Social and Community Medicine, University of Bristol, Canynge Hall, 39 Whatley Road, Bristol, BS8 2PS, UK

[2]Address: Bristol Randomised Trials Collaboration, University of Bristol, Canynge Hall, 39 Whatley Road, Bristol, BS8 2PS, UK

[3]Address: The Hull York Medical School, University of York, Heslington, York, YO10 5DD, UK

[4]Address: Urology Department, Royal United Hospital, Combe Park, Bath BA1 3NG, UK

[5]Address: Nuffield Department of Surgical Sciences, University of Oxford, Oxford, OX3 7DQ, UK

Correspondence: Dr Chris Metcalfe, email: chris.metcalfe@bristol.ac.uk, telephone: 0117 928 7326, address: School of Social and Community Medicine, University of Bristol, Canynge Hall, 39 Whatley Road, Bristol, BS8 2PS, UK

Funding: Supported by Cancer Research UK and the UK Department of Health for the CAP trial (C11043/A4286, C18281/A8145, C18281/A11326, and C18281/A15064). The ProtecT trial is funded by the UK National Institute for Health Research (NIHR), Health Technology Assessment Programme (projects 96/20/06, 96/20/99).

Word count: 4001

For peer review only - http://bmjopen.bmj.com/site/about/guidelines.xhtml

ABSTRACT

Objectives: Cross-sectional studies suggest that around 6% of men undergo PSA testing each year in UK general practice. This longitudinal study aims to determine the cumulative testing pattern of men over a 10-year period and whether this testing can be considered equivalent to screening for prostate cancer.

Setting, participants and outcome measures: Patient-level data on PSA tests, biopsies and prostate cancer (PCa) diagnoses were obtained from the UK Clinical Practice Research Datalink (CPRD) for the years 2002 to 2011. The cumulative risks of PSA testing and of being diagnosed with PCa were estimated for the 10-year study period. Associations of a man's age, region and index of multiple deprivation (IMD) with the cumulative risk of PSA testing and PCa diagnosis were investigated. Rates of biopsy and diagnosis, following a high test result, were compared to those from the programme of PSA testing in the ProtecT study.

Results: The 10-year risk of exposure to at least one PSA test in men aged 45 to 69 years in UK general practice was 39.2% (95% C.I. 39.0, 39.4%). The age-specific risks ranged from: 25.2% for 45-49 year olds to 53.0% for 65-69 year olds (P for trend<0.001). For those with a PSA level ≥3, a test in UK general practice was less likely to result in a biopsy (6%) and/or diagnosis of prostate cancer (15%) compared to ProtecT study participants (85% and 34% respectively).

Conclusion: A high proportion of 45-69 year old men undergo PSA tests in UK general practice: 39% over a ten year period. A high proportion of these tests appear to be for the investigation of lower urinary tract symptoms, and not screening for prostate cancer.

Trial registration: The ProtecT trial is registered at Current Controlled Trials (ISRCTN20141297) and Clinical Trials.Gov (NCT02044172).

For peer review only - http://bmjopen.bmj.com/site/about/guidelines.xhtml

STRENGTHS AND LIMITATIONS OF THIS STUDY

- This is the first study in the UK to look at patterns of PSA testing over a ten-year period in a cohort of men.
- Data on over 430,000 men could be analysed from the Clinical Practice Research Database and compared to data on 58,500 men from the programme of PSA testing and diagnostic biopsy in the ProtecT study

- The completeness of some routine data items is uncertain; with the recorded diagnoses outnumbering the recorded biopsies indicating that the latter are under-recorded
- It was not possible to distinguish tests undertaken in men with and without symptoms therefore the proportion of tests prompted by the presentation of LUTS was inferred.

For peer review only

For peer review only - http://bmjopen.bmj.com/site/about/guidelines.xhtml

INTRODUCTION

The UK currently runs three screening programmes for breast, bowel and cervical cancers. Prostate cancer is now the most commonly diagnosed cancer in men in the UK despite there being no formal screening programme.[1] Prostate Specific Antigen (PSA) level can be used as a screening test, with prostate biopsy in men with a raised PSA level allowing histopathological confirmation of the diagnosis of prostate cancer. Despite almost 30 years of PSA testing, the balance of benefits and harms of the test has not been established and, perhaps as a consequence, there are varying rates of testing around the UK and the world.[2] There is evidence that a PSA-based screening programme will reduce mortality due to prostate cancer[3] but with a risk of over diagnosis, such that a man diagnosed with cancer localised to the prostate would not have developed clinical symptoms of the disease in his lifetime if left untreated.[4 5] Radical treatment of such men exposes them to the risk of treatment-related adverse events without the potential to benefit.[6]

Current guidance for Primary Care Physicians in the UK, US and Australia recommends discussing and coming to a shared decision about PSA testing,[7] with men who either raise the issue or warrant consideration of testing, due to a family history of the disease for example. With such passive advice, variable testing rates across GPs are unsurprising. Three cross sectional studies have been conducted giving an indication of the PSA testing rates in the UK between 2001 and 2011. Melia et al., studying 469,159 men aged 45 to 84 years, reported an annual rate of 6% over 1999-2002 for England and Wales, with an annual rate of 2% in the absence of symptoms.[8] Williams et al., studying 126,716 men aged 45 to 89 years and without a prior diagnosis of prostate cancer, found 6.2% of these men received a PSA test during 2007.[9] This study concluded that testing was more prevalent in older men, more southern areas of the UK (especially Wales) and areas of lower deprivation. Moss et al. obtained data from the Clinical Practice Research Datalink on 650,264 men aged 45 to 84 and found a testing rate of 8.74 and 9.45 per 100 person-years in 2010 and 2011 respectively.[10] Again, rates increased with age and areas of lower deprivation. Of 49,306 men tested in 2010 and with at least 9 months of follow-up, 0.2% with a PSA level <3ng/ml were diagnosed with prostate cancer within 9 months, rising to 14.5% of men with PSA level >5ng/ml. A London-based study of 150,481 men aged 40

For peer review only - http://bmjopen.bmj.com/site/about/guidelines.xhtml

years or older found that 8.2% of men were PSA tested at their General Practice in the 12 months from August 2013 to July 2014.[11]

When PSA tests are undertaken for screening, men with a raised level will be referred for biopsy, with examination of prostate tissue necessary for the diagnosis of prostate cancer. Furthermore, as screen-detected prostate cancer is relatively slow to progress, screening is targeted at men in their fifties and sixties, the balance of risks of short-term treatment harms and longer-term survival benefit being less favourable for older men as death due to other causes is more likely and radical treatments less suitable. Tests which are unlikely to be followed-up by biopsy, and which are undergone by older men, are likely to be guiding the treatment of benign hyperplasia of the prostate.[12] Guidance for the assessment of lower urinary tract symptoms (LUTS), affecting approximately 30% of over 50s,[13] includes consideration of a PSA when LUTS are suggestive of bladder outlet obstruction secondary to benign prostatic enlargement; where PSA>1.4ng/ml can direct drug treatment decisions.[14]

While estimates of the number of men undergoing a PSA test in a twelve-month period give an indication of how widespread use of the test has become in UK general practice, a longitudinal perspective is needed to examine how the PSA test is being used to manage the risk of prostate cancer in individual men. Long term retrospective cohort studies of PSA testing rates have been conducted elsewhere in Europe;[15 16] however, the cumulative risks of PSA testing in the UK are yet to be quantified.

The primary objective of this study was to estimate the cumulative risk of PSA testing of UK men in primary care, without a diagnosis of prostate cancer, over the 10-year period 1st January 2002 to 31st December 2011. The association of testing rates with age, region and index of multiple deprivation (IMD) was investigated. The proportion of tests resulting in a biopsy and/or diagnosis of prostate cancer was compared to the programme of PSA testing, akin to screening, in the ProtecT study[17] to gauge whether PSA tests undertaken in UK General Practice can be considered as an effective attempt at screening.

For peer review only - http://bmjopen.bmj.com/site/about/guidelines.xhtml

SUBJECTS AND METHODS

Design

We undertook a retrospective cohort study of 450,000 men using data from the Clinical Practice Research Datalink (CPRD), a large primary care database.[18] The CPRD contains electronic medical records for approximately 4.4 million active patients in 674 practices, representing 6.9% of the UK population. Patients in the database were shown to be representative of the UK population in terms of age, sex, ethnicity and BMI. However, the data do not include prisoners, private patients, some residential homes and the homeless.[19] Practices participating in the CPRD have been found to have a greater number of patients compared to the national average.[20]

Data were requested for General Practice (GP) surgeries in all areas of UK, but excluding London as it is thought that PSA testing rates would be markedly different in the capital.[11] We included practices which contributed acceptable 'research standard' data for the observation period, 1st of January 2000 - 31st December 2011.[19] Data requested from the CPRD included: age, Index of Multiple Deprivation (IMD) from 2004, region, GP practice size, mortality date and cause, occurrence of PSA tests and prostate biopsies. PSA test dates before 2002 were also collected to estimate how many of the men had received a test prior to registration. The Index of Multiple Deprivation (IMD) is an area based deprivation measure which ranges from 0 to 100 with higher scores indicating higher levels of deprivation. CPRD base these on the patients' postcode (English residents only) and then create twentiles to ensure concealment of individuals' place of residence.

Study population

Entry to the cohort commenced on the 1st January 2002. Person-years for the time before the first PSA test were calculated having censored men from the analysis at the earliest of: (1) the end of the study period (31st December 2011); (2) after receiving a prostate cancer diagnosis; or (3) death or transfer out of the practice. Men aged 45 to 69 at study entry were included (those born between 1933 and 1957).

Practices thought to be involved with research involving practice-wide PSA testing within the eligible age group were excluded. For example, the ProtecT study[17] was recruiting at UK general practices

For peer review only - http://bmjopen.bmj.com/site/about/guidelines.xhtml

during 2001 to 2009. This exclusion was done by calculating the PSA testing rate for the men in each practice for all 60 two-month periods within the observation period, and excluding a practice if in any two-month period all the following conditions were satisfied: (1) the testing rate was >3.5 SDs higher than the overall practice average; (2) more than 10 men were tested; (3) more than 5% of men not previously tested were tested in this period.

Statistical Analysis

The follow-up period for each man was calculated as the difference in years between registration start date (on or after the 1st January 2002) and censoring (defined above). The Kaplan-Meier failure function estimated the cumulative proportion of men exposed to at least one PSA test, and diagnosed with prostate cancer over the course of the 10-year period for all men. The log-rank test was then used to investigate relationships between characteristics of the men and risk of undergoing a PSA test. A Cox Proportional Hazards model and Wald test were also used to check that associations remained, with or without accounting for clustering by practice. For men with full ten-year follow-up and no diagnosis of prostate cancer (before or during follow-up), logistic and ordinal logistic regression were used to explore relationships between age group and the number of tests each man received.

The percentage of men retested within 365 days of their first test was explored by age category and PSA level for the CPRD data. For this analysis, all men in CPRD who had 365 days of follow-up post PSA test were included; as well as those diagnosed within the 365 days. Associations between age and retesting were investigated using logistic regression. Serum PSA levels for both the first and second test were used to determine the percentage of men diagnosed out of those who were retested, given their first and second PSA levels. Those known to have had a PSA test but no level recorded were assumed to have undetectable levels (<0.1) and therefore equivalent to zero.

Data on men who attended PSA screening as part of the ProtecT study[17] were used to explore how routine data on PSA tests compare with the tests carried out as part of a screening intervention. The ProtecT data were divided between men with LUTS (lower urinary tract symptoms) and no LUTS and compared with the CPRD dataset, by age group and further broken down by PSA level. LUTS was defined using 5 questions from the International Continence Society Male Short-Form (ICSmaleSF)

For peer review only - http://bmjopen.bmj.com/site/about/guidelines.xhtml

questionnaire.[21] Men were classified as having LUTS if any of the following were true: (1) urinating every two hours or more during the day; (2) urinating at least twice during night; or (3) suffering ‘sometimes’, ‘most of the time’ or ‘all of the time’ from delayed urination, rushing to urinate or leaking before reaching the toilet. Men diagnosed with prostate cancer before having their first PSA test in ProtecT were removed and, as with CPRD, those with a PSA test date but no level recorded were assumed to be undetectable and therefore equivalent to zero. PSA level was broken down into the following categories to ensure adequate numbers in each group: PSA<3, 3≤PSA<4, 4≤PSA<6, 6≤PSA<10 and PSA≥10.

As an additional exploratory analysis, the percentage of men undergoing prostate biopsy and percentage diagnosed with prostate cancer within 365 days of their PSA test is also presented for men in the CPRD dataset and the two groups of ProtecT participants (LUTS and no LUTS). Comparisons between cohorts, and between risk groups within a cohort, were made using logistic and ordinal logistic regression. Within CPRD, biopsies and diagnoses were detected using medcodes provided by CPRD which correspond to Read-codes which are used in General Practice in the UK. Lists used are in the Supplementary material, Table S1.

The CPRD group holds ethical approval from a National Research Ethics Service Committee (NRECS) for all purely observational research using anonymised CPRD data. The ProtecT trial holds ethics approval from the Trent Multicentre Research Ethics Committee (Trent MREC), 21/06/2001, ref: 01/4/025.

For peer review only - http://bmjopen.bmj.com/site/about/guidelines.xhtml

RESULTS

Final cohort

In total, 450,000 men from 578 primary care practices across all regions of the UK (excluding London) were included in the CPRD data extract. Of these 450,000 men: 14 were removed due to missing or conflicting data; 303 were listed as having died before 2002; 2,184 had a diagnosis of prostate cancer date before 2002; and 369 patients had no follow up. From the remaining 447,130 patients, 12,894 (3%) men were removed as they were attendees of 19 practices suspected of participating in research involving practice-wide PSA testing. After the removal of these practices, the final sample was 434,236 men from 558 practices. Of these, 161,478 (37%) had the full 10-year follow up.

Risk of PSA testing and PCa diagnosis

The men were followed up for a cohort total of 2,963,645 person-years (median 8.25 years, IQR 3.83-10.00). Between 2002 and 2011 inclusively, 120,697 (28%) men received at least one PSA test and 7,538 (2%) men received a prostate cancer diagnosis. The cumulative 1, 5 and 10-year risks of receiving a PSA test were 5.1% (95% C.I. 5.0 to 5.2%), 21.4% (95% C.I. 21.3 to 21.5%) and 39.2% (95% C.I. 39.0 to 39.4%) respectively. The Kaplan-Meier curve illustrates the cumulative risk of PSA testing over the 10-year period, along with the age-specific cumulative risks (*Figure 1a*). A similar trend was seen in prostate cancer diagnoses (*Figure 1b*). The cumulative 1, 5 and 10 year risks of receiving a prostate cancer diagnosis were 0.2% (95% C.I. 0.2 to 0.2%), 1.0% (95% C.I. 1.0 to 1.1%) and 2.7% (95% C.I. 2.7 to 2.8%) respectively.

Table 1 shows the risks by age group, region, IMD quartiles and testing history. The risk of receiving a PSA test for men in the lowest age category (45-49 years) was substantially lower than the highest age category (65-69 years), with 10-year risks of exposure to PSA testing of 25.2% and 53.0% respectively (P<0.001). Likewise, the risk of diagnosis was also lower, with 10-year risks of 0.5% and 6.3% for age groups (45-49 years) and (65-69 years) respectively.

For peer review only

Table 1. Factors that influence the risk of having a PSA test/PCa diagnosis

		PSA testing			PCa diagnosis		
	N (%)	Men who had at least 1 PSA test	10 year risk % (95% C.I)£	P value	Men who had a PCa diagnosis	10 year risk % (95% C.I)£	P value
All men	434,236 (100%)	120,697	39.19 (39.01, 39.38)		7,538	2.72 (2.66, 2.78)	
**Age (in 2002)**							
45-49	104,782 (24%)	17,297	25.20 (24.86, 25.55)		296	0.49 (0.44, 0.55)	
50-54	100,211 (23%)	24,162	34.70 (34.33, 35.08)		858	1.40 (1.31, 1.50)	
55-59	97,224 (22%)	30,328	43.08 (42.69, 43.47)	p<0.001*	1,700	2.76 (2.63, 2.90)	p<0.001*
60-64	71,637 (17%)	25,518	48.57 (48.11, 49.04)		2,179	4.67 (4.47, 4.87)	
65-69	60,381 (14%)	23,392	52.95 (52.44, 53.45)		2,505	6.28 (6.04, 6.53)	
**Region**							
South East Coast	51,494 (12%)	17,434	47.45 (46.90, 48.01)		998	3.14 (2.95,3.34)	
Wales	35,277 (8%)	12,119	45.02 (44.40, 45.66)		689	2.79 (2.59, 3.01)	
Northern Ireland	12,730 (3%)	4,515	43.69 (42.70, 44.69)		264	2.75 (2.44, 3.11)	
South Central	53,577 (12%)	16,383	42.45 (41.93, 42.98)		976	2.79 (2.62, 2.98)	
South West	44,060 (10%)	12,399	40.82 (40.22, 41.42)		777	2.96 (2.75, 3.18)	
West Midlands	40,677 (9%)	11,453	39.28 (38.69, 39.88)	p<0.001$	704	2.66 (2.47, 2.86)	p<0.001$
North West	56,484 (13%)	16,340	38.88 (38.39, 39.37)		994	2.54 (2.38, 2.70)	
East of England	47,851 (11%)	12,386	38.85 (38.26, 39.44)		810	2.88 (2.68, 3.09)	
Yorkshire & the Humber	18,717 (4%)	4,131	35.49 (34.51, 36.50)		251	2.40 (2.10, 2.75)	
East Midlands	19,539 (5%)	4,466	34.43 (33.50, 35.38)		260	2.40 (2.10, 2.75)	
North East	8,113 (2%)	1,859	30.49 (29.31, 31.71)		123	2.25 (1.88, 2.69)	
Scotland	45,717 (11%)	7,212	23.82 (23.31, 24.33)		692	2.39 (2.21, 2.58)	
**Index of Multiple Deprivation (quartiles)**							
1-5 (Least deprived)	84,706 (20%)	29,422	46.26 (45.84, 46.67)		1,824	3.20 (3.06, 3.36)	
6-10	69,496 (16%)	21,611	42.45 (42.00, 42.91)		1,332	2.92 (2.76, 3.08)	
11-15	56,865 (13%)	14,596	36.32 (35.82, 36.82)	p<0.001*	916	2.54 (2.38, 2.72)	p<0.001*
16-20 (Most deprived)	40,833 (9%)	8,735	31.92 (31.33, 32.51)		483	1.92 (1.75, 2.10)	
No IMD recorded	182,336 (42%)	46,333	36.87 (36.58, 37.16)		2,983	2.63 (2.53, 2.73)	
**Pre-registration PSA test**							
Previously tested	27,211 (6%)	15,368	73.21 (72.54, 73.89)	p<0.001$	1,089	5.94 (5.59, 6.31)	p<0.001$
Not previously tested	407,025 (94%)	105,329	36.97 (36.78, 37.16)		6,449	2.51 (2.45, 2.57)	

£Kaplan Meier failure function at 10 years, *P for trend, $P across categories

For peer review only - http://bmjopen.bmj.com/site/about/guidelines.xhtml

PSA testing and diagnosis risks varied by region (p<0.001). The risks of testing and diagnosis were higher in more southern areas, especially the South East Coast (47.5% and 3.1% respectively) and Wales (45.0% and 2.8%). The lowest risks were found in Scotland (23.8% and 2.4%) and the North East (30.5% and 2.3%). Those living in areas of greater deprivation had a lower risk of testing (46.3% vs. 31.9%) and diagnosis (3.2% vs. 1.9%); p for trend<0.001.

Those who had received a PSA test prior to registration were substantially more likely to receive a PSA test and diagnosis than those who had not (73.2 vs 37.0 and 5.9 vs. 2.5 respectively); p<0.001.

Number of PSA tests

There were 157,586 men with complete 10-year follow-up and no prostate cancer. Of these, 57,491 men (36%) underwent at least one PSA test. Older age group was strongly related to a greater number of tests over 10 years (p<0.001, Table 2).

PSA levels and retesting

Data on PSA levels in CPRD were incomplete, but the median first PSA result of those tested with a result (n=119,175) was 1.23ng/ml (IQR=0.70-2.60; *Figure 2*). Assuming that those with a PSA test date but missing level (n=1,522, 1%) were undetectable and therefore ≈0, the median PSA result would be 1.20ng/ml (IQR=0.70-2.60). Removing the lowest age category (45-49) increased the median PSA to 1.30ng/ml (0.70-2.81), n=103,400. For the ProtecT men, the median PSA result of those collected (n=58,542) was 0.99ng/ml (IQR=0.60, 1.70) which remained the same after making the same assumption for the 27 men with a missing PSA result.

Of those PSA tested with a full year's follow up after their test (aged 50-69), 17,757/90,252 (20%) had a second test within a year of their first (Table 3). Undergoing a second PSA test within a year of the first test was strongly associated with a higher PSA level at the first test (OR per PSA category higher 1.83, 95% C.I. 1.80, 1.85; p<0.001). Those men with a PSA<3ng/ml were more likely to be retested within a year if they were in an older age group (OR per age category older 1.04, 95% confidence interval 1.04, 1.04; p<0.001). This trend was reversed for those men with a PSA≥3ng/ml

For peer review only - http://bmjopen.bmj.com/site/about/guidelines.xhtml

Table 2. Number of PSA tests received by men with full 10 year follow up and no prostate cancer diagnosis

Number of tests	0	1	2	3	4	5	6	7	8	9	≥10
All men	100,095 (64%)	28,561 (18%)	12,196 (8%)	6,047 (4%)	3,449 (2%)	2,050 (1%)	1,379 (1%)	929 (1%)	734 (<1%)	539 (<1%)	1,607 (1%)
**Age at entry in 2002**											
45-49	28,998 (77%)	5,651 (15%)	1,744 (5%)	647 (2%)	284 (1%)	162 (<1%)	81 (<1%)	37 (<1%)	23 (<1%)	20 (<1%)	47 (<1%)
50-54	25,299 (68%)	6,846 (18%)	2,549 (7%)	1,132 (3%)	606 (2%)	324 (1%)	195 (1%)	107 (<1%)	100 (<1%)	63 (<1%)	150 (<1%)
55-59	21,471 (59%)	7,092 (20%)	3,176 (9%)	1,654 (5%)	866 (2%)	549 (2%)	384 (1%)	246 (1%)	195 (1%)	144 (<1%)	380 (1%)
60-64	13,903 (54%)	4,941 (19%)	2,508 (10%)	1,371 (5%)	917 (4%)	524 (2%)	354 (1%)	277 (1%)	212 (1%)	160 (1%)	482 (2%)
65-69	10,424 (50%)	4,031 (19%)	2,219 (11%)	1,243 (6%)	776 (4%)	491 (2%)	365 (2%)	262 (1%)	204 (1%)	152 (1%)	548 (3%)

For peer review only

Table 3. PSA levels* by age group in the CPRD data and Protect men (LUTS vs. no LUTS)

	Age group 50-54			Age group 55-59			Age group 60-64			Age group 65-69		
PSA level	CPRD[$]	LUTS[£]	No LUTS[£]	CPRD[$]	LUTS[£]	No LUTS[£]	CPRD[$]	LUTS[£]	No LUTS[£]	CPRD[$]	LUTS[£]	No LUTS[£]
**Number of men tested (%)**												
PSA<3[~]	17898 (87%)	5228 (95%)	10178 (96%)	21183 (80%)	5716 (90%)	10331 (92%)	16306 (73%)	4730 (83%)	7560 (86%)	13622 (66%)	3646 (78%)	4681 (82%)
3≤PSA<4	936 (5%)	138 (3%)	216 (2%)	1714 (6%)	276 (4%)	410 (4%)	1734 (8%)	412 (7%)	469 (5%)	1776 (9%)	393 (8%)	394 (7%)
4≤PSA<6	838 (4%)	79 (1%)	137 (1%)	1655 (6%)	201 (3%)	290 (3%)	1868 (8%)	262 (5%)	407 (5%)	2026 (10%)	331 (7%)	340 (6%)
6≤PSA<10	500 (2%)	29 (1%)	61 (1%)	1092 (4%)	98 (2%)	145 (1%)	1340 (6%)	174 (3%)	193 (2%)	1642 (8%)	201 (4%)	188 (3%)
10≤PSA<20	294 (1%)	13 (<1%)	16 (<1%)	541 (2%)	29 (<1%)	41 (<1%)	717 (3%)	65 (1%)	96 (1%)	925 (4%)	88 (2%)	77 (1%)
PSA≥20	165 (1%)	3 (<1%)	7 (<1%)	350 (1%)	15 (<1%)	21 (<1%)	513 (2%)	28 (<1%)	39 (<1%)	614 (3%)	38 (1%)	52 (<1%)
**Number retested within 1 year of their first PSA test (%)**												
PSA<3[~]	1707 (10%)			2391 (11%)			2294 (14%)			2130 (16%)		
3≤PSA<4	256 (27%)			336 (20%)			308 (18%)			359 20%)		
4≤PSA<6	507 (61%)			902 (55%)			927 (50%)			819 (40%)		
6≤PSA<10	336 (67%)			681 (62%)			778 (58%)			934 (57%)		
10≤PSA<20	185 (63%)			315 (58%)			408 (57%)			489 (53%)		
PSA≥20	82 (49%)			163 (47%)			215 (42%)			235 (38%)		
**Number biopsied within 1 year of their first PSA test (%)**												
PSA<3[~]	20 (<1%)	0 (0%)	0 (0%)	36 (<1%)	0 (0%)	0 (0%)	28 (<1%)	0 (0%)	0 (0%)	28 (<1%)	0 (0%)	1 (<1%)
3≤PSA<4	13 (1%)	122 (88%)	174 (80%)	7 (<1%)	245 88%)	347 (83%)	10 (1%)	345 (84%)	374 (81%)	5 (<1%)	316 (81%)	307 (78%)
4≤PSA<6	61 (7%)	74 (94%)	122 (88%)	92 (6%)	186 (92%)	254 (86%)	87 (5%)	240 (90%)	339 (84%)	50 (3%)	278 (85%)	274 (81%)
6≤PSA<10	51 (10%)	27 (93%)	56 (92%)	133 (12%)	92 (91%)	134 (92%)	128 (10%)	160 (93%)	166 (86%)	139 (9%)	168 (84%)	161 (87%)
10≤PSA<20	36 (12%)	13 (93%)	16 (100%)	84 (16%)	26 (90%)	41 (98%)	109 (15%)	60 (94%)	89 (92%)	116 (13%)	81 (92%)	65 (87%)
PSA≥20	18 (11%)	3 (100%)	5 (71%)	56 (16%)	11 (69%)	17 (81%)	62 (12%)	23 (82%)	33 (87%)	66 (11%)	30 (81%)	39 (75%)
**Number diagnosed within 1 year of their first PSA test - with or without biopsy (%)**												
PSA<3[~]	18 (<1%)	0 (0%)	2 (<1%)	52 (<1%)	1 (<1%)	2 (<1%)	55 (<1%)	2 (<1%)	1 (<1%)	64 (<1%)	2 (<1%)	1 (<1%)
3≤PSA<4	19 (2%)	28 (20%)	47 (22%)	25 (2%)	62 (22%)	102 (24%)	18 (1%)	98 (24%)	115 (25%)	10 (1%)	87 (22%)	111 (28%)
4≤PSA<6	80 (10%)	29 (37%)	44 (32%)	124 (8%)	64 (32%)	95 (32%)	129 (7%)	80 (30%)	139 (34%)	79 (4%)	90 (28%)	119 (35%)
6≤PSA<10	99 (20%)	15 (52%)	27 (44%)	186 (17%)	37 (37%)	66 (45%)	258 (19%)	70 (41%)	96 (49%)	264 (16%)	74 (37%)	84 (45%)
10≤PSA<20	87 (30%)	6 (43%)	12 (75%)	155 (29%)	18 (62%)	29 (69%)	242 (34%)	35 (55%)	76 (78%)	296 (32%)	49 (56%)	47 (63%)
PSA≥20	86 (51%)	3 (100%)	7 (100%)	220 (63%)	11 (69%)	16 (76%)	347 (68%)	23 (82%)	34 (89%)	421 (69%)	32 (86%)	42 (81%)

*Those without a test date could not be included as we could not determine whether they had a full years follow up post-test, ~Those with a PSA test but missing PSA level were assumed to have a score that was undetectable and therefore below 3, $Data taken between Jan 2002-Dec 2011 for PSA tests taken in Jan 2002-Dec 2010 - any men without 1 full years follow up post-test were removed, £Data taken from the ProtecT study [17] between Jan 2002-Jan 2010 for PSA tests taken from Jan 2002-Jan 2009

For peer review only - http://bmjopen.bmj.com/site/about/guidelines.xhtml

where those in an older age group were less likely to be retested within a year than those in a younger age group (OR per age category older 0.98, 95% C.I. 0.97, 0.98; p<0.001).

Older men were also at greater risk of having a higher PSA test than those in younger age categories, for ProtecT and CPRD data, (for the CPRD cohort, OR per age group older 1.08, 95% C.I. 1.07, 1.08; p<0.001). On average, those ProtecT participants presenting with LUTS appeared to have higher PSA levels than those with no LUTS, while men in CPRD had the highest PSA results (*Table 3).*

Subsequent biopsies and diagnoses

From the ProtecT data, 22,200 men were identified as having LUTS (based on our definition) at the consultation for their PSA test and 36,364 men did not have LUTS. For men with a PSA level of 3ng/ml or higher, biopsy and diagnosis rates were much higher in ProtecT participants than CPRD. This remained true, even when those with a high PSA level were confirmed high in a further test (*Table S2*). Furthermore, for men in the CPRD cohort, a lower proportion underwent biopsy than were subsequently diagnosed. Overall, the odds of diagnosis within a year of a PSA test was 3 times higher in the ProtecT study compared with the CPRD data for those with PSA≥3, (OR 2.99, 95% C.I. 2.80, 3.18; p<0.001).

For CPRD, as expected, men with higher PSA level were more likely to be diagnosed (OR per PSA category higher 3.46, 95% C.I. 3.37, 3.56; p<0.001), as were older men (OR per age category higher 1.08, 95% C.I. 1.07, 1.08; p<0.001). For those aged between 50 and 69, the biopsy rates were <1%, 1%, 5%, 10% and 13% for PSA categories PSA<3, 3≤PSA<4, 4≤PSA<6, 6≤PSA<10 and PSA≥10 respectively. The diagnosis rates were <1%, 1%, 6%, 18% and 59% respectively.

For peer review only - http://bmjopen.bmj.com/site/about/guidelines.xhtml

DISCUSSION

This paper has examined the risk of receiving a PSA test over a 10-year period in a large retrospective cohort of men aged 45-69 in the UK (excluding London). The 10-year risk of undergoing a PSA test was estimated at 39.2% while the 10-year risk of receiving a prostate cancer diagnosis was estimated at 2.7%. Higher rates of both testing and diagnoses were associated with older age, more southerly region of residence, less deprived index of multiple deprivation (IMD), and a history of PSA testing. For all age groups and PSA levels, the proportion of men undergoing biopsy, and subsequently diagnosed with prostate cancer following a PSA test in UK general practice is low when compared to men in the PSA testing programme undertaken as part of the ProtecT trial.[17]

Overall the number of men without a prior diagnosis of prostate cancer receiving at least one PSA test over 10-years is high, especially given the lack of a screening programme in the UK. The higher rates of testing in older men is consistent with the findings of other studies of UK general practice[8-10] although not with the age-distribution of men agreeing to participate in the ProtecT study, the latter being in close agreement with the male population age distribution, with the majority of men being younger than 60 years.[17] These findings suggest that interest in prostate cancer screening is not concentrated in the older age groups, and that the greater incidence of testing in older men is likely to arise due to other diagnostic indications for the PSA test. The increase in testing with age could be due to an increase in lower urinary tracts symptoms with age[22] or with the GP wanting to rule out the possibly of prostate cancer[23] despite this rarely being the cause of such symptoms.[21 24] It is also thought that the PSA level is a useful indicator of prostate volume and may inform the choice between treatment options for BPH and other benign conditions.[12 25]

The observation of greater testing of men living in more affluent areas is consistent with previous studies.[8-10] This association presumably arises from more affluent men being more likely to request a test, or through general practices serving more affluent areas being more likely to promote the test to their male patients. There is some evidence to suggest that prostate cancer is more prevalent in areas of lower deprivation;[26] however, the extent to which PSA testing patterns inform this is difficult to determine.

For peer review only - http://bmjopen.bmj.com/site/about/guidelines.xhtml

Overall, 11% of men with 10 years of follow-up were tested three or more times. This varied by age group, 20% of men aged 65 to 69 years at the outset being tested three or more times, compared to 3% of men aged 45 to 49 years. Two major trials of prostate cancer screening have employed repeated PSA testing,[27 28] although the association with age would not be expected if the programmes in those trials were being followed by UK general practitioners, and certainly not a greater number of older men undergoing multiple tests.

ProtecT participants with LUTS had slightly higher PSA levels on average than those without LUTS. Whilst 78% of men in UK general practice undergoing a PSA test were found to have a level below 3ng/ml, on average PSA levels were higher than seen in the ProtecT study. In part this will be due to the older age profile of men in UK general practice compared to ProtecT participants, but it is also consistent with more tests in General Practice being undertaken to inform a diagnosis of LUTS, as LUTS are associated with elevated PSA levels (there is no reason to suppose a higher prevalence of non-symptomatic and undiagnosed prostate cancer in CPRD and ProtecT general practices). A strong association was observed for all age groups between higher PSA levels at a first test and the probability of a man undergoing a second test within one year, indicating that the results of the PSA tests did inform clinical management.

The incidence of biopsy and prostate cancer diagnosis in the CPRD cohort suggests that a PSA of 4ng/ml or more was being used in UK general practice as a trigger for further diagnostic investigations. The incidence of biopsy in the CPRD cohort is very low, and the fact that there are fewer biopsies than prostate cancer diagnoses suggests that either many more men were refusing biopsy, perhaps due to the full screening process not being discussed at the time of PSA testing, or that biopsy is being under-recorded in general practice data. However, even allowing for a degree of under-reporting, only a small minority of men with high PSA levels were recorded as having had a biopsy, which contrasts with 80% plus of ProtecT men with PSA of 3ng/ml or higher undergoing the investigation. Furthermore, the risk of a prostate cancer diagnosis in the CPRD cohort is much lower than in comparable men participating in the ProtecT study prospective PSA testing programme. These findings are again consistent with the majority of PSA tests in UK general practice being undertaken

For peer review only - http://bmjopen.bmj.com/site/about/guidelines.xhtml

to inform the diagnosis and management of LUTS in older men, with no intention of screening for prostate cancer.

Strengths and Limitations

The major strength of this investigation is the use of CPRD data which has allowed a large retrospective cohort of men to be constructed, and followed-up for a period of up to ten years. The use of CPRD data is also behind the key weakness of this study: the completeness of some data items is uncertain, with the recorded diagnoses outnumbering the recorded biopsies indicating that the latter are under-recorded, presumably even when cancer is diagnosed.

We did not attempt to distinguish those tests undertaken in men presenting with and without symptoms, and this could be considered a further limitation of our study. Screening aims to diagnose a disease before symptoms arise. However, prostate cancer rarely results in LUTS and sexual symptoms until it is at an advanced stage. For the vast majority of men with urinary and sexual symptoms the cause is benign, and in fact men with an elevated PSA are less likely to be diagnosed with prostate cancer if they also have LUTS or impaired sexual function.[21 29] The pattern of PSA testing in the CPRD cohort suggests that many PSA tests are being undertaken to inform the diagnosis and management of LUTS, and knowing which men had been PSA tested because of a presentation with symptoms would have lent further support to this hypothesis.

For peer review only - http://bmjopen.bmj.com/site/about/guidelines.xhtml

CONCLUSION

In UK general practice, 39.2% of men aged 45 to 69 years and initially free of prostate cancer undergo at least one PSA test during a 10-year follow-up period (2002 to 2011). However, testing rates are higher in the older age groups, and high PSA levels are commonly not followed up by a biopsy, required for the diagnosis of prostate cancer. Hence it is likely that a high proportion of these tests are related to investigations or management of lower urinary tract symptoms and other benign conditions, and cannot be considered as part of an effective (informal) effort to screen for prostate cancer.

For peer review only

AUTHOR CONTRIBUTIONS

ELT, SEO, YBS, JAL, DEN, FCH, JLD, RMM and CM contributed to the design of the study and formulated the research question. SH, ELT, EIW, RMM and CM contributed to the acquisition of the data. GJY, SH, ELT, EIW, SE, RMM and CM made substantial contributions to the analysis and interpretation of data for the work. GJY, SH and CM wrote the first draft. All authors commented on the drafts and revised it critically for important intellectual content. All authors approved the final manuscript and are accountable for the accuracy and integrity of the work.

ACKNOWLEDGEMENTS

We acknowledge the contribution of the CAP trial group. Investigators: RMM (Lead PI), JLD (PI), DEN (PI), FCH (PI), ELT (trial Co-ordinator), CM (statistician), Jonathan Sterne (statistician), Sian Noble (health economist), JAL. Research staff: Elizabeth Hill, Siaw Yien Ng, Naomi Williams, Liz Down (data manager), EIW (data manager), GJY (statistician), Joanna Thorn (health economist), Charlotte Davies, Laura Hughes, Mari-Anne Rowlands and Lindsey Bell. Management committee: ELT (Chair), RMM, JLD, CM, Jonathan Sterne, Sian Noble, YBS, JAL, SEO, Peter Brindle and SE. Trial steering committee: Michael Baum (Chair), Peter Albertsen, Tracy Roberts, Mary Robinson, Jan Adolfsson, David Dearnaley, AZ, Fritz Schröder, Tim Peters, Peter Holding, Teresa Lennon, Sue Bonnington, Malcolm Mason, Jon Oxley, RMM, JLD, DEN, FCH, ELT and JAL. Data monitoring committee: Lars Holmberg (Chair), Robert Pickard, Simon Thompson and Usha Menon. Cause of Death Committee: Peter Albertsen (Chair), Colette Reid, John McFarlane, Jon Oxley, Mary Robinson, Jan Adolfsson, Michael Baum, Anthony Zietman, Amit Bahl and Anthony Koupparis. Administrative staff: Marta Tazewell and Genevieve Hatton-Brown. We wish to extend our thanks to Pete Shiarly for the development of bespoke databases. We also wish to acknowledge the contribution of all members of the ProtecT study research groups. Thanks are extended to the Cancer Registries and staff at the Health and Social Care Information Centre. We acknowledge the contributions of the ProtecT and CAP study participants, investigators, researchers, data monitoring committees, and trial steering committees.

For peer review only

SUPPORT AND FUNDING

This work was supported by Cancer Research UK and the UK Department of Health for the CAP trial (C11043/A4286, C18281/A8145, C18281/A11326, and C18281/A15064). CAP is sponsored by the University of Bristol and is registered at Current Controlled Trials (ISRCTN92187251). The ProtecT trial is funded by the UK National Institute for Health Research (NIHR), Health Technology Assessment Programme (projects 96/20/06, 96/20/99). The views and opinions expressed herein are our own and do not necessarily reflect those of the Department of Health. The ProtecT trial is sponsored by the University of Oxford and is registered at Current Controlled Trials (ISRCTN20141297) and Clinical Trials.Gov (NCT02044172).

We acknowledge the support from the Oxford NIHR Biomedical Research Centre through the Surgical Innovation and Evaluation Theme, and the Surgical Interventional Trials Unit, and Cancer Research UK through the Oxford Cancer Research Centre.

SH is a Wellcome Trust-funded PhD student with Grant Code 102432/Z/13/Z. JLD is supported, in part, by the NIHR Collaboration for Leadership in Applied Health Research and Care West, hosted by University Hospitals Bristol NHS Foundation Trust. FCH is supported, in part, by the Oxford NIHR Biomedical Research Centre and the Cancer Research UK Oxford Centre. RMM is supported, in part, by University Hospitals Bristol NHS Foundation Trust National Institute for Health Research Bristol Nutrition Biomedical Research Unit and CRUK (C18281/A19169). JLD, FCH and DEN are NIHR Senior Investigators. The views and opinions expressed herein are the authors own and do not necessarily reflect those of the Department of Health. The funders and sponsor had no role in the design and conduct of the study, or the preparation of the report or decision to publish. The corresponding author had full access to the data and takes responsibility for the decision to submit for publication.

CONFLICT OF INTEREST

The authors declare no conflict of interest.

For peer review only - http://bmjopen.bmj.com/site/about/guidelines.xhtml

DATA SHARING

The authors do not have permission to share data obtained from the Clinical Practice Research Datalink (CPRD). Baseline data from the ProtecT trial may be available on application (email info-protect@bris.ac.uk).

For peer review only

For peer review only - http://bmjopen.bmj.com/site/about/guidelines.xhtml

REFERENCES

1. Office for National Statistics [ONS]. Cancer Registration Statistics, England: 2015 [Available from: https://www.ons.gov.uk/peoplepopulationandcommunity/healthandsocialcare/conditionsanddiseases/bulletins/cancerregistrationstatisticsengland/2015 accessed 7 July 2017.
2. Roobol MJ. The prostate-specific antigen test. *Expert Opin Med Diagn* 2013;7(5):423-6. doi: 10.1517/17530059.2013.821980
3. Schroder FH, Hugosson J, Roobol MJ, et al. Screening and prostate cancer mortality: results of the European Randomised Study of Screening for Prostate Cancer (ERSPC) at 13 years of follow-up. *Lancet* 2014;384(9959):2027-35. doi: 10.1016/S0140-6736(14)60525-0
4. Chou R, Croswell JM, Dana T, et al. Screening for Prostate Cancer: A Review of the Evidence for the US Preventive Services Task Force. *Ann Intern Med* 2011;155(11):762-U94. doi: 10.7326/0003-4819-155-11-201112060-00375
5. Gulati R, Mariotto AB, Chen S, et al. Long-term projections of the harm-benefit trade-off in prostate cancer screening are more favorable than previous short-term estimates. *J Clin Epidemiol* 2011;64(12):1412-17. doi: 10.1016/j.jclinepi.2011.06.011
6. Donovan JL, Hamdy FC, Lane JA, et al. Patient-Reported Outcomes after Monitoring, Surgery, or Radiotherapy for Prostate Cancer. *New Engl J Med* 2016;375(15):1425-37. doi: 10.1056/NEJMoa1606221
7. Pickles K, Carter SM, Rychetnik L. Doctors' approaches to PSA testing and overdiagnosis in primary healthcare: a qualitative study. *BMJ Open* 2015;5(3) doi: ARTN e00636710.1136/bmjopen-2014-006367
8. Melia J, Moss S, Johns L. Rates of prostate-specific antigen testing in general practice in England and Wales in asymptomatic and symptomatic patients: a cross-sectional study. *BJU International* 2004;94(1):51-56. doi: 10.1111/j.1464-410X.2004.04832.x
9. Williams N, Hughes LJ, Turner EL, et al. Prostate-specific antigen testing rates remain low in UK general practice: a cross-sectional study in six English cities. *BJU International* 2011;108(9):1402-08. doi: 10.1111/j.1464-410X.2011.10163.x
10. Moss S, Melia J, Sutton J, et al. Prostate-specific antigen testing rates and referral patterns from general practice data in England. *Int J Clin Pract* 2016;70(4):312-18. doi: 10.1111/ijcp.12784
11. Nderitu P, Van Hemelrijck M, Ashworth M, et al. Prostate-specific antigen testing in inner London general practices: are those at higher risk most likely to get tested? *BMJ Open* 2016;6(7) doi: ARTN e01135610.1136/bmjopen-2016-011356
12. Roehrborn CG, McConnell JD, Lieber M, et al. Serum prostate-specific antigen concentration is a powerful predictor of acute urinary retention and need for surgery in men with clinical benign prostatic hyperplasia. *Urology* 1999;53(3):473-80. doi: Doi 10.1016/S0090-4295(98)00654-2
13. National Institute of Health and Care Excellence [NICE]. LUTS in men 2015 [Available from: https://cks.nice.org.uk/luts-in-men#!topicsummary accessed 14 March 2017.
14. National Institute for Health and Care Excellence [NICE]. Lower urinary tract symptoms in men: management 2010 [Available from: https://www.nice.org.uk/guidance/cg97/chapter/1-Recommendations accessed 14 March 2017.
15. Nordstrom T, Aly M, Clements MS, et al. Prostate-specific Antigen (PSA) Testing Is Prevalent and Increasing in Stockholm County, Sweden, Despite No Recommendations for PSA Screening: Results from a Population-based Study, 2003-2011. *European Urology* 2013;63(3):419-25. doi: 10.1016/j.eururo.2012.10.001
16. Hamoen EHJ, Reukers DFM, Numans ME, et al. Discrepancies between guidelines and clinical practice regarding prostate-specific antigen testing. *Family Practice* 2013;30(6):648-54. doi: 10.1093/fampra/cmt045
17. Lane JA, Donovan JL, Davis M, et al. Active monitoring, radical prostatectomy, or radiotherapy for localised prostate cancer: study design and diagnostic and baseline results of the ProtecT randomised phase 3 trial. *Lancet Oncology* 2014;15(10):1109-18. doi: 10.1016/S1470-2045(14)70361-4
18. Walley T, Mantgani A. The UK General Practice Research Database. *Lancet* 1997;350(9084):1097-99. doi: Doi 10.1016/S0140-6736(97)04248-7

For peer review only - http://bmjopen.bmj.com/site/about/guidelines.xhtml

19. Herrett E, Gallagher AM, Bhaskaran K, et al. Data Resource Profile: Clinical Practice Research Datalink (CPRD). *International Journal of Epidemiology* 2015;44(3):827-36. doi: 10.1093/ije/dyv098
20. Campbell J, Dedman DJ, Eaton SC, et al. Is the CPRD GOLD Population Comparable to the UK Population? *Pharmacoepidemiology and Drug Safety* 2013;22:280-81.
21. Collin S, Metcalfe C, Donovan J, et al. Associations of lower urinary tract symptoms with prostate-specific antigen levels and screen-detected localized and advanced prostate cancer: A case-control study nested within the UK population-based protect (prostate testing for cancer and treatment) study. *Neurourology and Urodynamics* 2008;27(7):583-84.
22. Andersson SO, Rashidkhani B, Karlberg L, et al. Prevalence of lower urinary tract symptoms in men aged 45-79 years: a population-based study of 40,000 Swedish men. *BJU International* 2004;94(3):327-31. doi: 10.1111/j.1464-410X.2004.04930.x
23. Young JM, Muscatello DJ, Ward JE. Are men with lower urinary tract symptoms at increased risk of prostate cancer? A systematic review and critique of the available evidence. *BJU International* 2000;85(9):1037-48. doi: DOI 10.1046/j.1464-410x.2000.00659.x
24. Martin RM, Vatten L, Gunnell D, et al. Lower urinary tract symptoms and risk of prostate cancer: The HUNT 2 Cohort, Norway. *International Journal of Cancer* 2008;123(8):1924-28. doi: 10.1002/ijc.23713
25. Speakman MJ, Kirby RS, Joyce A, et al. Guideline for the primary care management of male lower urinary tract symptoms. *BJU International* 2004;93(7):985-90. doi: 10.1111/j.1464-410X.2004.04765.x
26. National Cancer Registration and Analysis Service. Cancer by Deprivation in England 1996 - 2011 2014 [Available from: http://www.ncin.org.uk/about_ncin/cancer_by_deprivation_in_england accessed 14 March 2017.
27. Schroeder FH, Hugosson J, Roobol MJ, et al. Screening and Prostate-Cancer Mortality in a Randomized European Study. *New Engl J Med* 2009;360(13):1320-28. doi: 10.1056/NEJMoa0810084
28. Andriole GL, Grubb RL, Buys SS, et al. Mortality Results from a Randomized Prostate-Cancer Screening Trial. *New Engl J Med* 2009;360(13):1310-19. doi: 10.1056/NEJMoa0810696
29. Collin SM, Metcalfe C, Donovan J, et al. Associations of Sexual Dysfunction with Localized and Advanced Prostate Cancer: A Case-Control Study Nested within the Uk Population-Based Protect (Prostate Testing for Cancer and Treatment) Study. *Eur Urol Suppl* 2009;8(4):139-39.

For peer review only - http://bmjopen.bmj.com/site/about/guidelines.xhtml

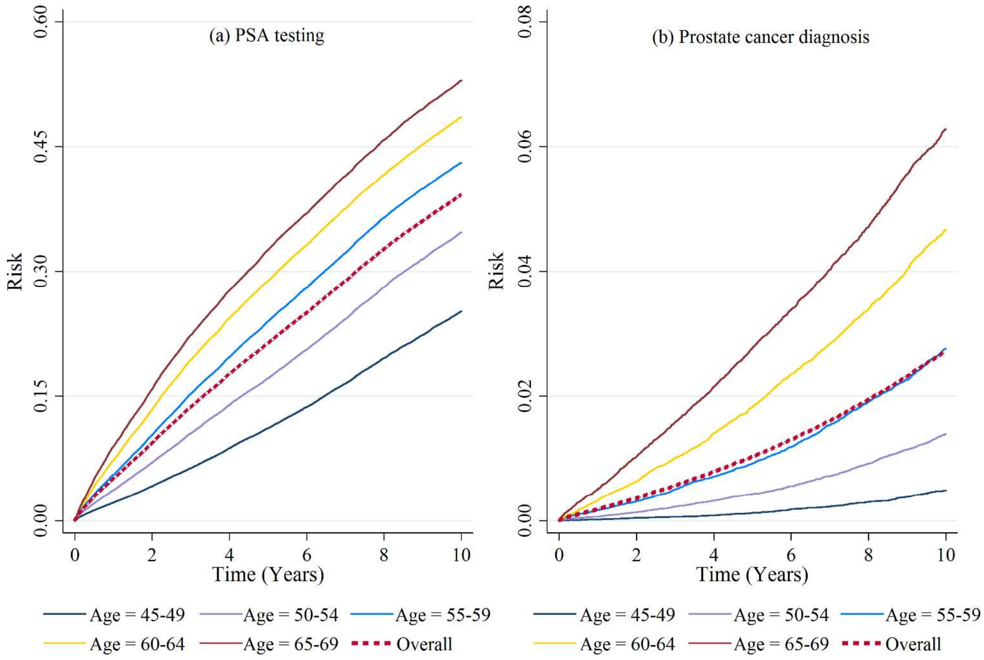

Figure 1a. Kaplan Meier failure estimate: Cumulative risk over 10 years of receiving a PSA test, by age group, during the period 2002 to 2012‼ ┼ Figure 1b. Kaplan Meier failure estimate: Cumulative risk over 10 years of receiving a prostate cancer diagnosis, by age group, during the period 2002 to 2012

254x184mm (300 x 300 DPI)

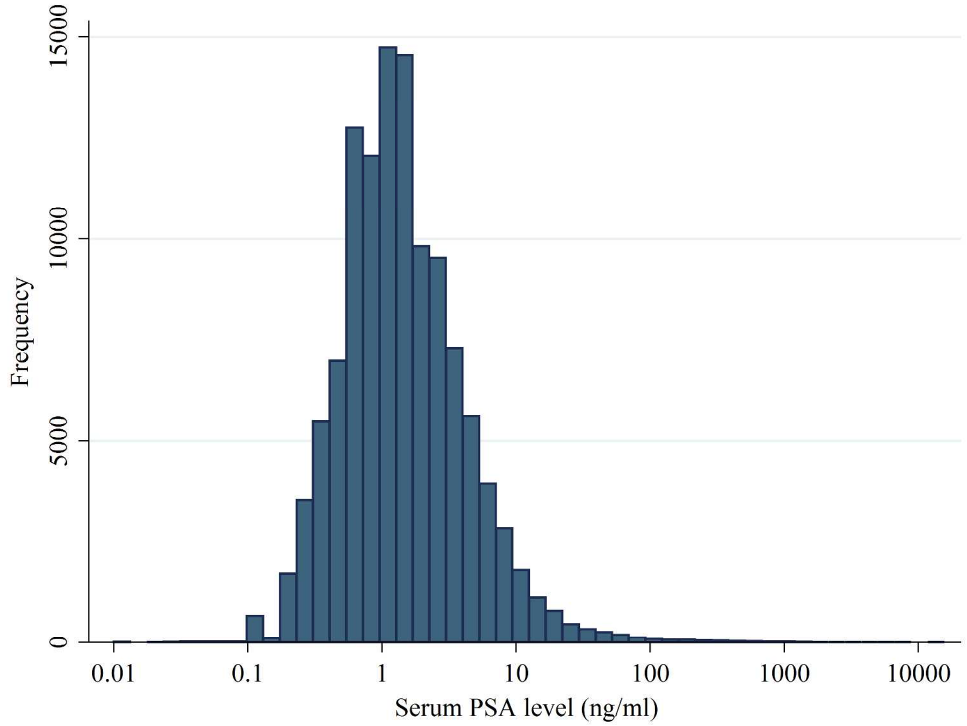

Figure 2. Distribution of PSA levels on the log scale

254x190mm (300 x 300 DPI)

ew only

Supplementary material

Table S1. List of used medical codes for prostate cancer biopsies and diagnoses

Biopsies		Diagnoses	
Medcode	Readterm	Medcode	Readterm
1069	*Transrectal needle biopsy of prostate*	780	*Malignant neoplasm of prostate*
7908	*Open biopsy of prostate*	6328	*Carcinoma in situ of prostate*
7909	*Unspec diagnostic cystoscopic exam bladder & biopsy prostate*	10178	*Gleason grading of prostate*
12391	*Transurethral biopsy prostate*	18503	*Gleason prostate grade 2-4 (low)*
22297	*Trucut transperineal biopsy of prostate*	18612	*Gleason prostate grade 5-7 (medium)*
22473	*Transperineal needle biopsy of prostate*	26081	*Gleason prostate grade 8-10 (high)*
22719	*Endoscopic punch biopsy of prostate*	37306	*Personal history of malignant neoplasm of prostate*
		102314	*History of prostate cancer*

Table S2. The percentage of men diagnosed with prostate cancer in CPRD based on their first and second PSA test

**% diagnosed (n/N)**	**Second test (within 1 year) PSA level**					
	No 2nd test	PSA<3~	PSA<3*	3≤PSA<6	6≤PSA<10	PSA≥10
**First test PSA level**						
PSA<3*	<1% (119/60487)	<1% (35/7692)	<1% (37/7876)	1% (6/477)	18% (17/97)	14% (10/72)
3≤PSA<6	3% (234/8133)	1% (6/748)	2% (14/850)	5% (162/3027)	14% (66/483)	15% (8/54)
6≤PSA<10	24% (452/1845)	1% (3/230)	6% (18/302)	4% (23/622)	17% (269/1556)	18% (45/249)
PSA≥10	61% (1232/2030)	6% (11/183)	14% (35/246)	3% (6/227)	9% (29/338)	43% (552/1281)

*PSA tests without a level recorded were assumed to be below the detection level and hence <3ng/ml
~Men with second PSA tests without a level recorded are not included

For peer review only

STROBE Statement—checklist of items that should be included in reports of observational studies

	Item No.	Recommendation	Check	Page No.
**Title and abstract**	1	(*a*) Indicate the study's design with a commonly used term in the title or the abstract	✓	1,2
		(*b*) Provide in the abstract an informative and balanced summary of what was done and what was found	✓	2
**Introduction**				
Background/rationale	2	Explain the scientific background and rationale for the investigation being reported	✓	4,5
Objectives	3	State specific objectives, including any prespecified hypotheses	✓	5
**Methods**				
Study design	4	Present key elements of study design early in the paper	✓	6
Setting	5	Describe the setting, locations, and relevant dates, including periods of recruitment, exposure, follow-up, and data collection	✓	6,7,8
Participants	6	(*a*) *Cohort study*—Give the eligibility criteria, and the sources and methods of selection of participants. Describe methods of follow-up<br>*Case-control study*—Give the eligibility criteria, and the sources and methods of case ascertainment and control selection. Give the rationale for the choice of cases and controls<br>*Cross-sectional study*—Give the eligibility criteria, and the sources and methods of selection of participants	✓	6,7,8
		(*b*) *Cohort study*—For matched studies, give matching criteria and number of exposed and unexposed<br>*Case-control study*—For matched studies, give matching criteria and the number of controls per case	N/A	-
Variables	7	Clearly define all outcomes, exposures, predictors, potential confounders, and effect modifiers. Give diagnostic criteria, if applicable	✓	6,7,8
Data sources/ measurement	8*	For each variable of interest, give sources of data and details of methods of assessment (measurement). Describe comparability of assessment methods if there is more than one group	✓	6,7,8
Bias	9	Describe any efforts to address potential sources of bias	N/A	-
Study size	10	Explain how the study size was arrived at	✓	6

Continued on next page

For peer review only - http://bmjopen.bmj.com/site/about/guidelines.xhtml

Quantitative variables	11	Explain how quantitative variables were handled in the analyses. If applicable, describe which groupings were chosen and why	✓	8
Statistical methods	12	(*a*) Describe all statistical methods, including those used to control for confounding	✓	7,8
		(*b*) Describe any methods used to examine subgroups and interactions	N/A	-
		(*c*) Explain how missing data were addressed	✓	8
		(*d*) *Cohort study*—If applicable, explain how loss to follow-up was addressed<br>*Case-control study*—If applicable, explain how matching of cases and controls was addressed<br>*Cross-sectional study*—If applicable, describe analytical methods taking account of sampling strategy	N/A	-
		(*e*) Describe any sensitivity analyses	N/A	-
**Results**				
Participants	13*	(a) Report numbers of individuals at each stage of study—eg numbers potentially eligible, examined for eligibility, confirmed eligible, included in the study, completing follow-up, and analysed	✓	9
		(b) Give reasons for non-participation at each stage	N/A	-
		(c) Consider use of a flow diagram	N/A	-
Descriptive data	14*	(a) Give characteristics of study participants (eg demographic, clinical, social) and information on exposures and potential confounders	✓	10
		(b) Indicate number of participants with missing data for each variable of interest	N/A	-
		(c) *Cohort study*—Summarise follow-up time (eg, average and total amount)	✓	9
Outcome data	15*	*Cohort study*—Report numbers of outcome events or summary measures over time	✓	9,10
		Case-control study—Report numbers in each exposure category, or summary measures of exposure	N/A	-
		Cross-sectional study—Report numbers of outcome events or summary measures	N/A	-
Main results	16	(*a*) Give unadjusted estimates and, if applicable, confounder-adjusted estimates and their precision (eg, 95% confidence interval). Make clear which confounders were adjusted for and why they were included	✓	9,10,11,14
		(*b*) Report category boundaries when continuous variables were categorized	✓	9,10,14
		(*c*) If relevant, consider translating estimates of relative risk into absolute risk for a meaningful time period	N/A	-

Continued on next page

For peer review only

For peer review only - http://bmjopen.bmj.com/site/about/guidelines.xhtml

Other analyses	17	Report other analyses done—eg analyses of subgroups and interactions, and sensitivity analyses	N/A	-
**Discussion**				
Key results	18	Summarise key results with reference to study objectives	✓	15,16
Limitations	19	Discuss limitations of the study, taking into account sources of potential bias or imprecision. Discuss both direction and magnitude of any potential bias	✓	3,17
Interpretation	20	Give a cautious overall interpretation of results considering objectives, limitations, multiplicity of analyses, results from similar studies, and other relevant evidence	✓	15,16,17,18
Generalisability	21	Discuss the generalisability (external validity) of the study results	✓	16,18
**Other information**				
Funding	22	Give the source of funding and the role of the funders for the present study and, if applicable, for the original study on which the present article is based	✓	1,20

*Give information separately for cases and controls in case-control studies and, if applicable, for exposed and unexposed groups in cohort and cross-sectional studies.

Note: An Explanation and Elaboration article discusses each checklist item and gives methodological background and published examples of transparent reporting. The STROBE checklist is best used in conjunction with this article (freely available on the Web sites of PLoS Medicine at http://www.plosmedicine.org/, Annals of Internal Medicine at http://www.annals.org/, and Epidemiology at http://www.epidem.com/). Information on the STROBE Initiative is available at www.strobe-statement.org.

For peer review only

For peer review only - http://bmjopen.bmj.com/site/about/guidelines.xhtml
